# Genome-wide Profiling of 8-Oxoguanine Reveals Its Association with Spatial Positioning in Nucleus

**DOI:** 10.1093/dnares/dsu023

**Published:** 2014-07-09

**Authors:** Minako Yoshihara, Li Jiang, Shinya Akatsuka, Mikita Suyama, Shinya Toyokuni

**Affiliations:** 1 Medical Institute of Bioregulation, Kyushu University, Maidashi 3-1-1, Higashi-ku, Fukuoka 812-8582, Japan; 2 Department of Pathology and Biology of Diseases, Graduate School of Medicine, Kyoto University, Kyoto 606-8501, Japan; 3 Department of Pathology and Biological Responses, Nagoya University Graduate School of Medicine, Tsurumai-cho 65, Showa-ku, Nagoya 466-8550, Japan; 4 Japan Science and Technology Agency, CREST, Fukuoka 812-8582, Japan

**Keywords:** 8-oxoguanine, lamina-associated domain, DNA modification, oxidative stress

## Abstract

8-Oxoguanine (8-oxoG) is one of the most common DNA lesions generated by reactive oxygen species. In this study, we analysed the genome-wide distribution profile of 8-oxoG by combining immunoprecipitation by antibodies specific for the DNA fragments containing 8-oxoG with a microarray that covers rat genome. Genome-wide mapping of 8-oxoG in normal rat kidney revealed that 8-oxoG is preferentially located at gene deserts. We did not observe differences in 8-oxoG levels between groups of genes with high and low expression, possibly because of the generally low 8-oxoG levels in genic regions compared with gene deserts. The distribution of 8-oxoG and lamina-associated domains (LADs) were strongly correlated, suggesting that the spatial location of genomic DNA in the nucleus determines the susceptibility to oxidative modifications. One possible explanation for high 8-oxoG levels in LADs is that the nuclear periphery is more susceptible to the oxidative damage caused by the extra-nuclear factors. Moreover, LADs have a rather compact conformation, which may limit the recruitment of repair components to the modified bases.

## 1. Introduction

Since the emergence of life on Earth, deoxyribonucleic acid, the carrier of genetic information, has been continuously exposed to various types of damaging factors, including internally generated reactive oxygen species (ROS), and several external factors, such as ionizing radiation, UV radiation, ROS, and other reactive molecules.^1^ Unrepaired DNA lesions cause mutations, which can lead to undesirable cell death, carcinogenesis, or age-related pathophysiology.^2^ Living organisms have various DNA repair systems to counteract these threats and to deal with errors in the DNA replication process. Thus, DNA repair systems are essential for all organisms, from bacteria to higher eukaryotes; most of these systems have been evolutionarily conserved.^3,4^

Because life evolved to use highly reactive molecular oxygen for energy production under aerobic conditions, ROS is one of the major factors involved in the generation of DNA lesions. Among oxidative DNA lesions, with more than 100 types of modifications identified in mammals, 8-oxoguanine (8-oxoG; also known as its tautomer 8-hydroxyguanine) is the most abundant and well-characterized base modification.^5,6^ It has been shown that, under normal conditions, there is one 8-oxoG molecule per 10^5^–10^6^ guanines in a genome, corresponding to thousands of 8-oxoG molecules per single cell.^7,8^

Genomic 8-oxoG can be formed in two ways; direct oxidation of a guanine residue in a genomic sequence or oxidation at the nucleotide pool levels, i.e. the formation of 8-oxo-dGTP followed by its incorporation during DNA synthesis.^9,10^ If a guanine residue in the genome is oxidized to 8-oxoG and the strand is used as a template in replication, an adenine residue is often incorporated in the daughter strand as the complementary nucleotide. This is because not only cytosine but also adenine can pair with 8-oxoG. As a consequence, G → T transversion mutation is frequently observed at the position of 8-oxoG modification.^9^ During replication, the oxidized nucleotide, 8-oxo-dGTP, may be incorporated into the complementary strand to pair with an adenine residue; in the next round of replication, cytosine may be inserted complementary to 8-oxoG. This would result in an A → C transversion mutation.^11^ It has been reported that G → T somatic mutation is mostly observed in oxidative stress-associated tumours such as lung, breast, ovarian, gastric, and colon cancers.^12,13^ It has also been reported that 8-oxoG modifications are associated with other diseases such as neurodegenerative and ageing-associated disorders.^14–17^

There are several methods to measure 8-oxoG contents in genomes. The total 8-oxoG has been quantified using the HPLC–ECD (electrochemical detection) system,^18^ and specific regions of several hundred bases have been analysed by ligation-mediated polymerase chain reaction (LM-PCR).^19,20^ There are also some reports of 8-oxoG measurements in entire genomes using fluorescent *in situ* hybridization (FISH-based)^21^ and DNA immunoprecipitation (IP) methods,^22^ although the obtained resolution of 8-oxoG along with the chromosomes by these methods is tens of Mb.

No detailed analysis of genome-wide distribution of DNA damages is available thus far.^23^ To examine the distribution of 8-oxoG modifications and identify susceptible regions in the genome with high resolution, we conducted array-based comprehensive profiling. The resulting 8-oxoG profile revealed that there is a clear negative correlation between 8-oxoG distribution and gene density. The negative correlation can be attributed to spatial positioning of chromosomes, so-called chromosome territory (CT)^24^ in the nucleus, where gene deserts tend to be located at perinucleic regions. Our results can be explained by the fact that perinucleic regions are more accessible to the external ROS than the regions close to the centre of the nucleus. Another possible explanation is that the chromosomes at perinucleic regions tend to be in compact conformations, preventing the recruitment of the 8-oxoG repair components.

## 2. Materials and methods

### 2.1. Samples

Male Wistar rats (specific pathogen-free; SPF), obtained from Japan SLC, Inc. (Shizuoka, Japan), were maintained in an SPF environment. The rats were sacrificed at 5 weeks of age, and the kidneys were harvested. The Institutional Animal Care and Use Committees of Kyoto University and Nagoya University approved all the animal experimentation protocols. The experiments were performed with two biological replicates.

### 2.2. Genomic DNA extraction and 8-oxoG immunoprecipitation

Fresh rat kidneys were frozen at −80°C, and 100 mg of the tissue was homogenized in 1.0 ml of homogenization buffer (0.15 M NaCl, 0.1 M EDTA; pH 8.0) with 0.1 mM desferal using a glass homogenizer on ice. Genomic DNA was extracted using the NaI method (Wako, Osaka, Japan); the solution was saturated with argon gas and contained 0.1 mM desferal to prevent further DNA oxidation. The DNA was digested with BmgT120I (G^GNCC) (Takara Bio, Shiga, Japan) at 37°C for 90 min. Fragment sizes were established by agarose gel electrophoresis (1.5% w/v agarose). The average size of the fragments was 500 bp.

The digested DNA samples were subjected to IP; 5 µg aliquots of DNA fragments were immunoprecipitated by adding 10 µg of 8-oxoG-specific monoclonal antibody, N45.1, purchased from Japan Institute for the Control of Aging (Shizuoka, Japan). The specificity of the antibody against the DNA fragments containing 8-oxoG was already validated by quantitative PCR analysis.^22,25^ IP was performed in 900 µl of the reaction buffer (0.1% bovine serum albumin, 10 mM phosphate buffer; pH 7.4), with stirring at 4 r.p.m. for 3 h at 4°C. Subsequently, 100 µl of Dynabeads (M-280 sheep anti-mouse IgG) (Dynal, Oslo, Norway) was added to the mixture (final volume, 1000 µl) and mixed at 4 r.p.m. for 5 h at 4°C. The Dynabeads complex was washed twice in the following three buffers: buffer 1: 0.1% sodium deoxycholate, 1% Triton X-100, 1 mM EDTA, 50 mM HEPES–KOH, 140 mM NaCl (pH 7.5); buffer 2: 0.1% sodium deoxycholate, 1% Triton X-100, 1 mM EDTA, 50 mM HEPES–KOH, 500 mM NaCl (pH 7.5); and buffer 3: 0.1% sodium deoxycholate, 0.5% Nonidet P-40, 1 mM EDTA, 250 mM LiCl, 10 mM Tris–HCl, (pH 8.0). The immunoprecipitated beads were eluted from the Dynabeads complex with 80 µl of elution buffer (10 mM EDTA, 1% sodium dodecyl sulphate, 50 mM Tris–HCl; pH 8.0) at 65°C for 10 min.

### 2.3. Microarray

For two-colour microarray, the test immunoprecipitated DNA fragments and 10 ng of input (control) DNA fragments (only digested by BmgT120I, without IP by N45.1 monoclonal antibody) were treated with proteinase K at 37°C for 1 h, followed by phenol–chloroform extraction and ethanol precipitation. The following oligonucleotides were used as adaptor DNA: 5′-GNCTGCGGTGA-3′ and 5′-AGCACTCTCCAGCCTCTCACCGCA-3′; complementary sequences are underlined. Ligations of the adaptor to the test and input DNA were performed using Ligation Pack (Nippon Gene, Toyama, Japan), following the standard protocol. The fragments with the adaptor were amplified with two PCR cycles.

The test and input DNA (800 ng per sample) were labelled with Cy5 and Cy3, respectively, using the standard protocol for Agilent DNA Labeling Kit (Agilent Technologies), and the mixed samples were applied to the Rat Genome CGH Microarray Kit 244A (Agilent Technologies), following the manufacturer's instructions. The arrays were scanned using the Agilent DNA Microarray Scanner G2505B (Agilent Technologies). The scanned data were subjected to image analysis using Agilent's Feature Extraction Software (v10.5.1.1; Agilent Technologies) with the default parameters. Two biologically replicated microarray experiments were performed.

### 2.4. Genome sequence data, annotations, and other publicly available data

The rat genome sequence data (assembly November 2004; rn4)^26^ and the associated annotation data were downloaded from the UCSC Genome Browser.^27^

To draw the distribution of 8-oxoG along chromosomes, we applied a smoothing method for each probe by calculating the averaged 8-oxoG level for all the probes in 500 kb in both directions.

To compare the distribution of 8-oxoG with gene expression data previously generated using microarrays (GSE7625),^28^ gene expression data for normal rat kidney (GSM184440 and GSM184441) were downloaded from GEO database at NCBI.^29^ The raw data were normalized using MAS5 package in Bioconductor/R.^30^ Subsequently, the normalized values were log_2_-transformed for both biological replicates.

The data for lamina-associated domains (LADs) and the DNA adenine methyltransferase identification (DamID) profiles of lamin B1 in mouse embryonic fibroblast^31^ were downloaded from UCSC Genome Browser.^27^ To compare these data with our 8-oxoG profile data for the rat, we converted the genomic coordinates using the liftOver program provided by UCSC Genome Browser.^27^ We set the -minBlocks option to 0.1 to reduce unconverted regions.

We used the custom track function of UCSC Genome Browser to visually examine the distribution of properties along the chromosomes. For detailed analysis and high-quality image generation over a wide range, we wrote graph-drawing programs in Perl, PostScript, and gnuplot.

### 2.5. Statistical analysis

We used the R package to draw boxplots, histograms, and contour plots. The R package was also used to calculate *P*-values for Student's *t*-tests.

## 3. Results

### 3.1. Genome-wide distribution of 8-oxoG

**Figure 1.**
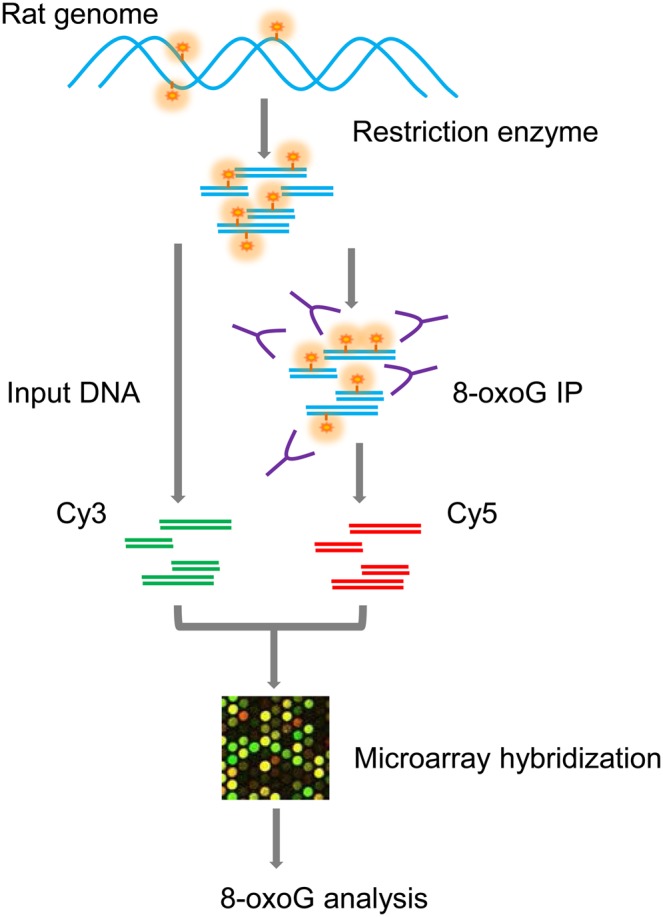
Schematic representation of the workflow for genome-wide detection of 8-oxoG. DNA double strands are shown in cyan; 8-oxoG residues are depicted in orange. The antibodies specific for the 8-oxoG-containing DNA are shown in purple. DNA obtained without immunoprecipitation was used as an input (control). Immunoprecipitated DNA and input DNA fragments were labelled with Cy5 and Cy3 dyes, respectively, and applied to the microarrays.

To obtain a complete distribution of 8-oxoG in normal rat kidney chromosomes, we performed DNA immunoprecipitation (IP) followed by microarray analysis using input (control) DNA fragments without IP as a reference (Fig. 1; for details, see the Materials and Methods section).

**Figure 2.**
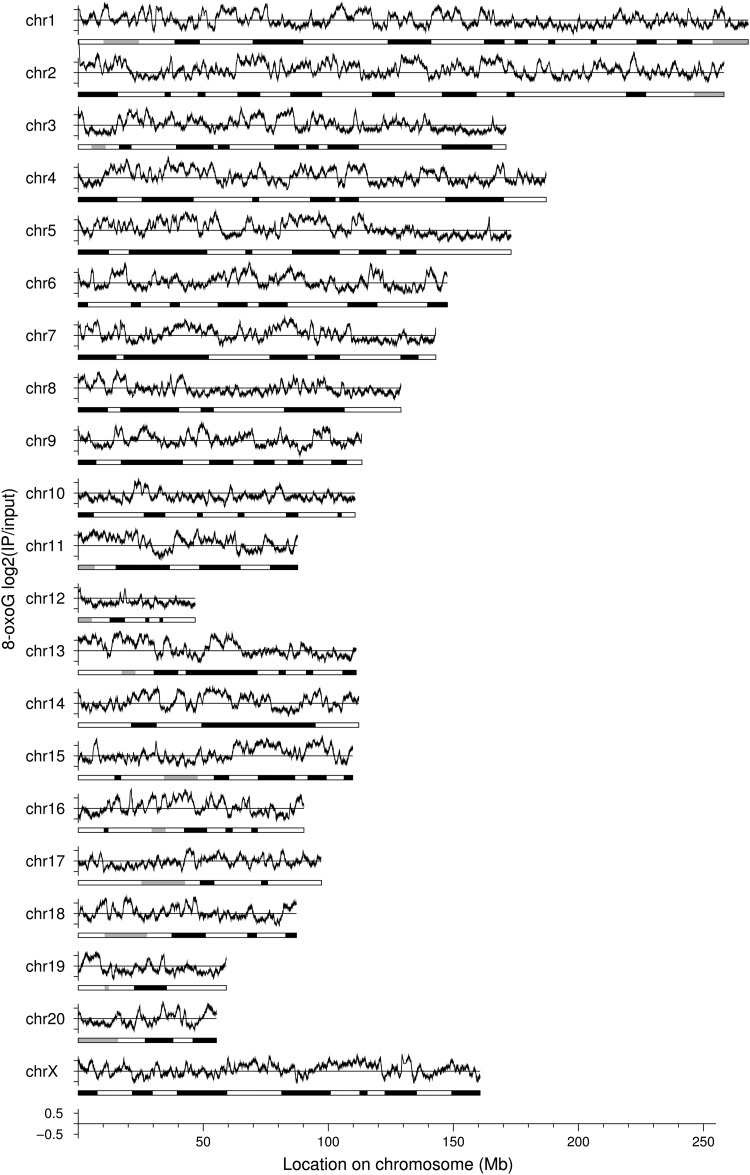
Distribution of 8-oxoG along the chromosomes. The vertical axis corresponds to log_2_ intensity of the array data. Distribution profile for 8-oxoG was calculated by averaging the intensities over a 1 Mb sliding window. Chromosome ideogram is shown under each plot.

To analyse the distribution of 8-oxoG, we plotted the signal of each probe of the microarray in the rat chromosomes. There are ∼244,000 probes (60-mer in length) on the microarray used in this study (Agilent Rat Genome CGH Microarray Kit 244A), corresponding to the average probe interval of 1 in 6,000 bases. The averaged 8-oxoG levels are plotted along the chromosomes and shown together with the ideogram in Figure 2. The plot reveals that the regions with high 8-oxoG level are not uniformly distributed; their size fluctuates around several Mb, with some 8-oxoG-rich regions larger than 10 Mb. For example, there is a broad peak spanning more than 10 Mb in the position of 50–60 Mb in chr14. However, there are also some 8-oxoG-depleted regions. We did not observe any clear correlation between the 8-oxoG levels and ideogram-banding patterns. The average probe interval of the microarray that we used in this experiment is 6,000 bases, while the 8-oxoG levels fluctuate with intervals of several Mb. This indicates that the probe density is high enough to depict the 8-oxoG profile along chromosomes.

**Figure 3.**
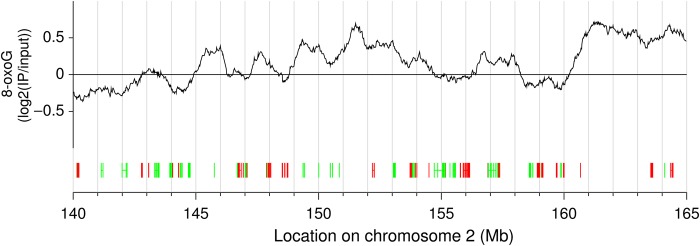
A representative genomic region showing 8-oxoG profile with gene annotation. The vertical axis corresponds to log_2_ intensity of the array data. The distribution profile of 8-oxoG was calculated by averaging the intensities over a 1 Mb sliding window. Gene structures are shown below the 8-oxoG profile. The directionality of the genes, i.e. forward and reverse orientation, is shown in green and red, respectively.

To explore the distribution of 8-oxoG along the chromosomes in detail, we enlarged a part of the plot, together with its gene annotations, in an arbitrarily selected genomic region (Fig. 3). A negative correlation between the 8-oxoG levels and gene location was observed, showing that 8-oxoG levels were lower in gene-rich regions than in gene deserts.

### 3.2. 8-oxoG distribution is negatively correlated with gene density

**Figure 4.**
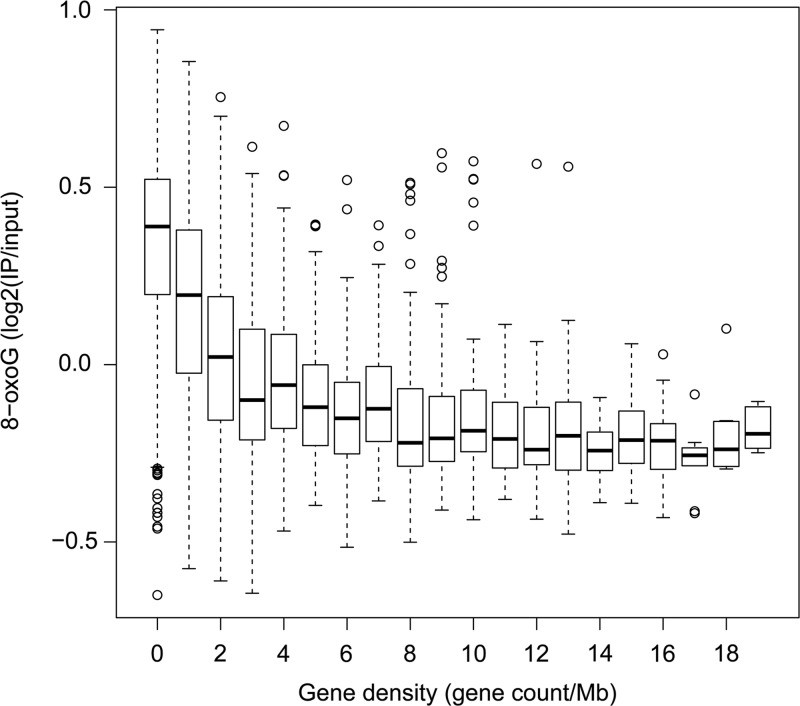
Box plot for the relationship between gene density (horizontal axis) and averaged 8-oxoG level (vertical axis). Chromosomes were divided into 1 Mb of non-overlapping regions, and the number of genes and averaged 8-oxoG levels were calculated for each region.

After the negative relationship between 8-oxoG distribution and gene density had been established by visual inspection of the distribution profiles and chromosome-scale quantification, we performed quantitative assessment of these relationships at a higher resolution over the entire genome. To achieve this, we divided the chromosomes into non-overlapping 1 Mb fragments, and counted the genes and averaged log_2_(IP/input) value of 8-oxoG for each fragment (Fig. 4). The box plot clearly shows that 8-oxoG levels increase with the decrease in the number of genes in a fragment (Pearson's correlation coefficient (*r*) = −0.47, *P* < 0.001), particularly for fragments with less than three genes. For the fragments without any genes (627 fragments), the median of averaged log_2_(IP/input) value of 8-oxoG is 0.39, while for the fragments with three genes (255 fragments), the averaged log_2_(IP/input) value for 8-oxoG level is −0.11. There are no clear differences between the values for 8-oxoG levels for the fragments with eight or more genes. This shows that the fragments with two or fewer genes tend to have more 8-oxoG than the fragments with three or more genes, confirming that the observed negative relationship between the profile for 8-oxoG levels and gene density was true on the genome-wide scale. In general, the GC content in gene deserts are lower than that in gene-rich regions, indicating that higher 8-oxoG levels in gene deserts are not simply due to biased base composition.

This trend can be partially validated by the independent data obtained in our previous study.^22^ By analysing these data, we have observed that the chromosomes with high 8-oxoG levels have low gene density, while those with high gene density have relatively low 8-oxoG levels. The data from that study were of low resolution compared with the results obtained in the present study. However, the fact that a negative correlation between 8-oxoG levels and gene density has been found in two independent studies gives more weight to our conclusions.

### 3.3. Lack of significant association between 8-oxoG levels and transcriptional activity

To analyse the negative correlation between 8-oxoG distribution and gene density, we examined the gene expression levels to establish whether the 8-oxoG levels in genic regions associates with transcriptional activity of the genes.

We used the gene expression data from microarray experiments in our previous study, which were performed on matched samples (GSM184440 and GSM184441).^28^ On the basis of the distribution of gene expression levels, we calculated the median expression value and used this value to divide the genes into two groups, i.e. those with high and low expression. For each gene, we calculated the averaged 8-oxoG level using log_2_(IP/input) values of the probes located within genic region, i.e. from the transcription start to end site. The values were subsequently used to draw histograms (Supplementary Fig. S1). The two distributions were almost identical; we did not observe any statistically significant differences (Student's *t*-test; *P* = 0.043), indicating that the 8-oxoG levels in genic regions do not associate with transcriptional activity of the genes. We also applied the same statistical test to the gene sets with extreme expression levels, i.e. (i) genes with top 25% and bottom 25% expression levels and (ii) genes with top 10% and bottom 10% expression levels, and compared the distributions of 8-oxoG levels between these groups of genes with the extreme expression. The former set (top/bottom 25%) gives *P* = 0.100 and the latter set (top/bottom 10%) gives *P* = 0.101, further confirming that the 8-oxoG levels in genic regions does not associate with transcriptional activity of the genes.

### 3.4. Association between 8-oxoG levels and structural features of chromosomes in the nucleus

It has recently been reported that the organization of chromosomes in the nucleus has a certain architecture, known as CT.^24^ CT is closely linked with gene regulation in mammalian cells and is conserved between species.^32,33^ The structural features of chromosomes have been demonstrated using FISH for specific loci^34^ and by DamID for LADs at a sub-megabase resolution.^31^ Lamins are filamentous proteins found in nuclear lamina at the nuclear periphery, serving as the scaffold of the nuclear envelope, and LADs are the specific chromosomal regions with higher AT content located close to the nuclear lamina. Gene density along the chromosomes is correlated with the structural features of chromosomes; gene-dense regions tend to be located towards the centre of the nucleus, while gene-poor regions are observed close to the nuclear periphery, i.e. LADs.

**Figure 5.**
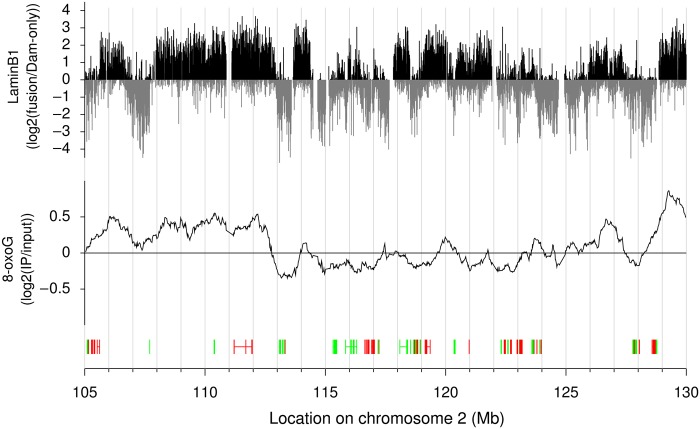
A representative genomic region showing 8-oxoG profile (middle track) and the profile of LADs (upper track). The profile of 8-oxoG was calculated by averaging the intensities over a 1 Mb sliding window. Gene structures are shown below the 8-oxoG profile. The directionality of the genes, i.e. forward and reverse orientation, is shown in green and red, respectively.

**Figure 6.**
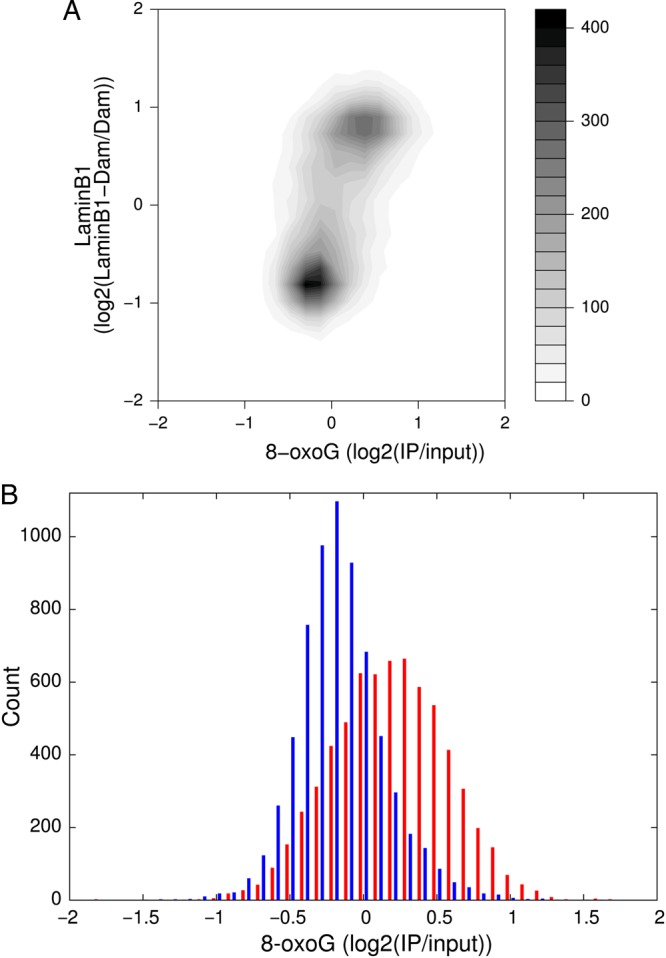
Correlation between 8-oxoG and LADs profiles. (A) A contour plot of averaged values of 8-oxoG and lamin B1 levels in 200 kb genomic fragments. (B) Histograms of averaged values of 8-oxoG in 200 kb genomic fragments. The fragments are grouped into two subsets using the averaged lamin B1 value of 0 as a cut-off. Sets with high and low contents of lamin B1 are shown in red and blue, respectively.

To analyse the distribution of 8-oxoG in terms of the three-dimensional architecture of chromosomes, we compared its distribution with the lamin B1 profile determined for the mouse cells using DamID mapping.^31^ There is no profile available for lamin B1 in the rat cells; however, because it has been shown that LADs do not considerably change between different cell types and species,^35^ we used the data for mouse embryonic fibroblasts. First, we downloaded the data of the lamin B1 profile for these fibroblasts with the coordinates on mm9 assembly and subsequently converted the coordinates to rat (rn4) using the liftOver program.^36^ The 8-oxoG and lamin B1 profiles showed a clear correlation (Fig. 5; Supplementary Fig. S2). For quantitative assessment of the correlation, we divided the genomic sequence into 200 kb fragments, and calculated the averaged 8-oxoG and lamin B1 values for each fragment. We adopted 200 kb as a fragment size because LADs generally span from 100 kb to 10 Mb.^37^ We converted the averaged values of these two indices for each 200 kb fragment into a contour plot to visually evaluate the relationship between 8-oxoG and lamin B1 profiles (Fig. 6A). We observed a positive trend between 8-oxoG and lamin B1 levels even though LADs are high AT content. The transitions between regions with low and high levels of lamin B1 are extremely sharp;^37^ we observed clear bimodal distribution in terms of lamin B1 levels. Next, following the bimodal distribution, the genomic fragments were grouped into two subsets using the averaged lamin B1 value of 0 as a cut-off, and the data were used to plot the histograms shown in Figure 6B. Averaged 8-oxoG values in the negative and positive lamin B1 groups were −0.13 and 0.25, respectively. The histograms show significantly different distributions (Student's *t*-test; *P* < 2.2 × 10^−16^), indicating that there is an excess of 8-oxoG in LADs.

To confirm the significance of this trend, we evaluated the chance to obtain such differences in 8-oxoG levels in negative and positive lamin B1 groups by randomly assigning lamin B1 levels to genomic fragments. We iterated this procedure 1,000 times and confirmed that such differences do not occur by chance (*z*-score = 55; *P* < 10^−10^).

To further confirm that the observed correlation is not due to an artefact of using lamin B1 profile obtained from mouse embryonic fibroblasts transferred to rat genome but irrespective of cell types, species, and fragment size for averaging, we conducted the same analysis by using lamin B1 data for the following samples: mouse astrocytes, mouse nueral progenitor cells, mouse embryonic stem cells, and human Tig3 lung fibroblasts.^31,37^ According to the previous paper,^35^ 71% of the genomic regions are similar among different cell types in terms of LAD localization. This can be divided into two parts: the genomic regions that are constitutively classified as LADs (constitutive LADs) (33%) and the genomic regions that are always LAD free (constitute inter-LADs) (38%). By transferring the data for each cell types to rat genome, and also by changing the fragment size to 50 and 500 kb, we always obtained similar patterns of distributions to that of mouse embryonic fibroblasts with the fragment size for averaging of 200 kb, indicating that the observation that the excess of 8-oxoG in LAD regions is not affected by the data source to be transferred to rat genome and the fragment size for averaging, but rather found to be a robust feature of 8-oxoG distribution (Supplementary Fig. S3).

## 4. Discussion

The first study of the genome-wide distribution of 8-oxoG has been performed for human lymphocytes using *in situ* immunodetection.^21^ The study has shown that 8-oxoG is unevenly distributed in the genome but follows a specific pattern; the distributions are similar in different individuals.^21^ In our previous study, we have successfully determined the distribution of 8-oxoG along chromosomes in mouse kidney cells at low resolution; we have used DNA immunoprecipitation with the 8-oxoG-specific antibody and cloning followed by Sanger sequencing.^22^ Both studies obtained low-resolution profiles (∼10 Mb). In the present study, we improved our previous procedure^22^ to achieve considerably higher resolution by adopting DNA immunoprecipitation followed by microarray analysis. Therefore, we obtained the 8-oxoG distribution along rat genome with the average probe interval of 6 kb.

There is a dynamic equilibrium between generation and repair of 8-oxoG modifications; therefore, the distribution observed is a snapshot of such an equilibrium state. The main 8-oxoG repair pathway is the base excision repair (BER). This repair pathway, which involves OGG1, MTH1, and MUTYH enzymes,^38^ exists in a wide range of species from prokaryotes to higher eukaryotes.^39^ Some studies have reported another type of 8-oxoG repair mechanism in *Escherichia coli*, a transcription-coupled repair (TCR)^40,41^ although 8-oxoG is a non-helix-distorting base lesion. Recently, it has been suggested that a similar repair system may also exist in eukaryotes.^42^ If such a system existed in eukaryotes, we would observe a negative correlation between 8-oxoG levels and transcriptional activity. However, we did not observe significant correlation between 8-oxoG levels and gene expression levels. This does not exclude the existence of TCR for 8-oxoG in eukaryotes because 8-oxoG levels in genic regions is rather low compared with gene-poor regions, which makes it difficult to detect subtle, if any, differences. If we increase the 8-oxoG level by artificial oxidative stress, we may be able to see a negative correlation between the 8-oxoG level and transcriptional activity.

**Figure 7.**
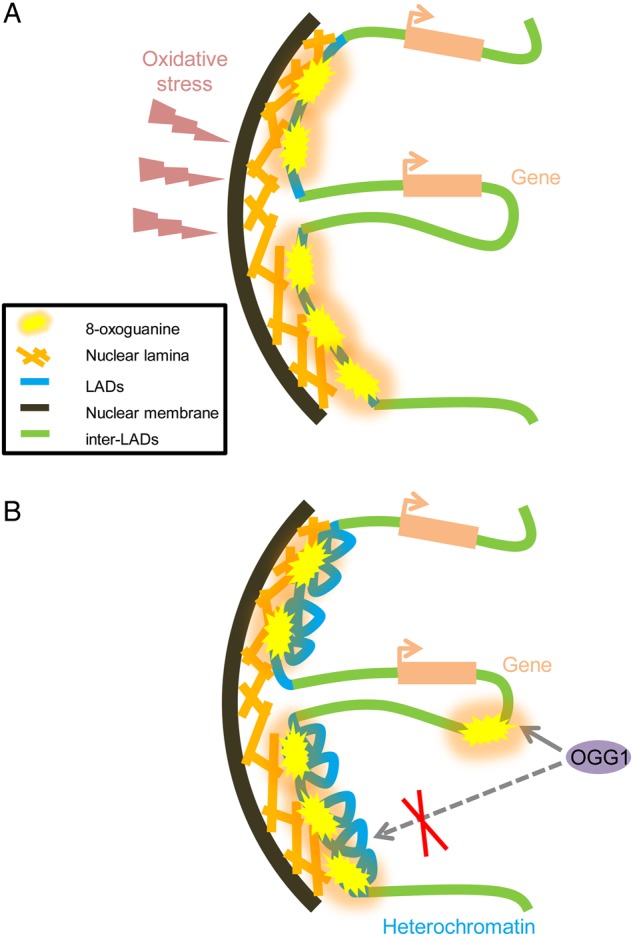
Two models showing the association of 8-oxoG distribution with the relative position in the nucleus. Marks used in the model are described in the panel. (A) Extra-nuclear oxidative stress mainly oxidizes the guanine residues in LADs. (B) Heterochromatin structures in LADs impede the access of repair enzymes, such as OGG1.

There are several possible reasons for the differences in 8-oxoG levels along chromosomes. According to the CT model, gene-rich regions tend to be located towards the interior of the nucleus, whereas gene-poor regions are closer to the nuclear periphery.^24^ The negative correlation observed between the 8-oxoG level and gene density may reflect the spatial architecture of chromosomes in the nucleus. LADs are well-characterized structural features of chromosomes.^31^ Therefore, we focused on the relationship between 8-oxoG and LADs. The distribution profiles of 8-oxoG and LADs are highly similar, suggesting that 8-oxoG is preferentially formed in LADs. This can be interpreted in at least two ways. One possible explanation is that LADs occupy the outermost regions of chromosomes in the nuclear spatial architecture. These regions are more exposed to the external oxidative stresses than the centrally located parts of chromosomes and more likely to be modified (Fig. 7A). This observation is consistent with the ‘bodyguard’ hypothesis proposed by Hsu.^43^ The author argued that heterochromatin localized at the nuclear periphery may protect the centrally located euchromatin against mutagens and other substances that modify chromosomal DNA. Although experimental evidence that directly supports this hypothesis was not provided for a long time,^34^ our observation that 8-oxoG tends to co-localize with LADs is in agreement with the hypothesis. In addition, guanine is known to be susceptible to oxidation because of its low oxidation potential, leading to a speculation that the formation of 8-oxoG may protect other genomic regions from mutagenesis due to oxidative stress.^44^

Another explanation of the similarity between 8-oxoG and LAD profiles is that the compact structure of LADs may prevent repair components from interacting with these regions (Fig. 7B). It has been shown that LADs are mostly inactive with regard to transcription and other cellular activities and are dominated by suppressive histone modifications such as H3K27me3.^37^ It has been also shown that LADs form heterochromatin.^45^ In heterochromatic regions, the recruitment of enzymes involved in BER, such as OGG1, is blocked; BER is less active in the heterochromatic than in euchromatic regions.^46^

There is a positive correlation between 8-oxoG levels, ageing, and carcinogenesis,^6^ which led us to focus on the detailed distribution of 8-oxoG along the chromosomes. We used normal rat kidney tissue, which is a suitable model for carcinogenesis due to oxidative stress. Rat kidney cancer induced by oxidative reagents, such as FeNTA, has long been used as a model for such cancers.^47,48^ In this model, the development of cancer takes from 1 to 2 years after injecting with FeNTA, which is comparable with the lifespan of a rat. The process resembles the development of cancer in humans to a larger extent than several transgenic mouse cancer models, which develops cancer considerably shorter period of time.^49–51^ Although the rate of cancer development is generally rather lower in rodents than in humans, rats injected with FeNTA develop cancer more frequently than mice after the same treatment.^47,52^ For these reasons, rats seem to be a model species particularly suitable for the analysis of cancer development in humans. We are planning to trace genome-wide changes in the distribution of 8-oxoG under oxidative stress condition to see if 8-oxoG increases in nuclear periphery. This will lead to further experiments about cancer development by measuring the 8-oxoG distributions at different stages of cancer to reveal its role in cancer progression. The data obtained in this study can constitute a basis for further studies in this and related fields.

## 5. Availability

Microarray data are available from the NCBI Gene Expression Omnibus (GEO) (http://www.ncbi.nlm.nih.gov/geo/) under accession number GSE54007.

## Supplementary data

Supplementary data are available at www.dnaresearch.oxfordjournals.org

## Funding

This work was supported in part by a grant-in-aid for research from the Ministry of Education, Culture, Sports, Science, and Technology (MEXT) of Japan (20017017 and 22132005 to M.S.; 221S0001-04 to S.T.) and Kyushu University Interdisciplinary Program in Education and Projects in Research Development (P&P) to M.S. Funding to pay the Open Access publication charges for this article was provided by the Ministry of Education, Culture, Sports, Science and Technology of Japan.

## Supplementary Material

Supplementary Data
