# Supplementary material for: Genome-wide Profiling of 8-Oxoguanine Reveals Its Association with Spatial Positioning in Nucleus
Source: DNA Res. 2014 Jul 9;21(6):603–12. doi: 10.1093/dnares/dsu023 (PMC4263294; doi:10.1093/dnares/dsu023)
Supplement: Supplementary Data [file supp_21_6_603__index.html]

Genome-wide Profiling of 8-Oxoguanine Reveals Its Association with Spatial Positioning in Nucleus — Genome-wide Profiling of 8-Oxoguanine Reveals Its Association with Spatial Positioning in Nucleus — Supplementary Data 

# Genome-wide Profiling of 8-Oxoguanine Reveals Its Association with Spatial Positioning in Nucleus

## Supplementary Data

Supplementary Data

**Files in this Data Supplement:**

- Supplementary Data - Pdf file
