# Supplementary material for: Genome-wide Profiling of 8-Oxoguanine Reveals Its Association with Spatial Positioning in Nucleus
Source: DNA Res. 2014 Jul 9;21(6):603–12. doi: 10.1093/dnares/dsu023 (PMC4263294; doi:10.1093/dnares/dsu023)
Supplement: Supplementary Data [file supp_dsu023_dsu023supp.pdf]

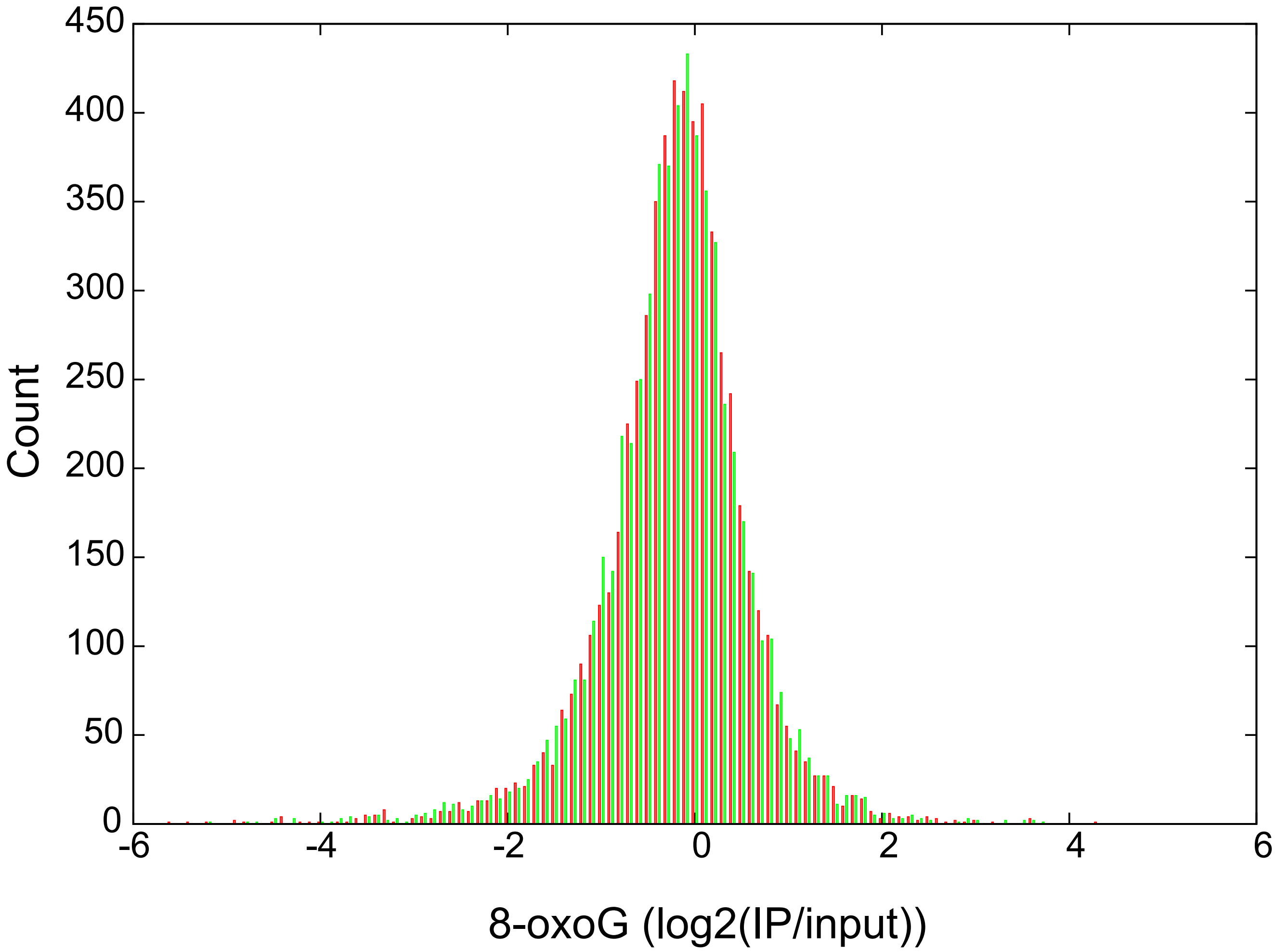


Figure S1. Histograms for the relationships between expression level of genes and 8-oxoG level. The horizontal axis represent averaged log2 intensity of 8-oxoG level measured by CGH array, and the vertical axis represent gene counts. Genes are divided into two groups, i.e., highly-expressed genes (in green) and the lowly-expressed genes (in red).

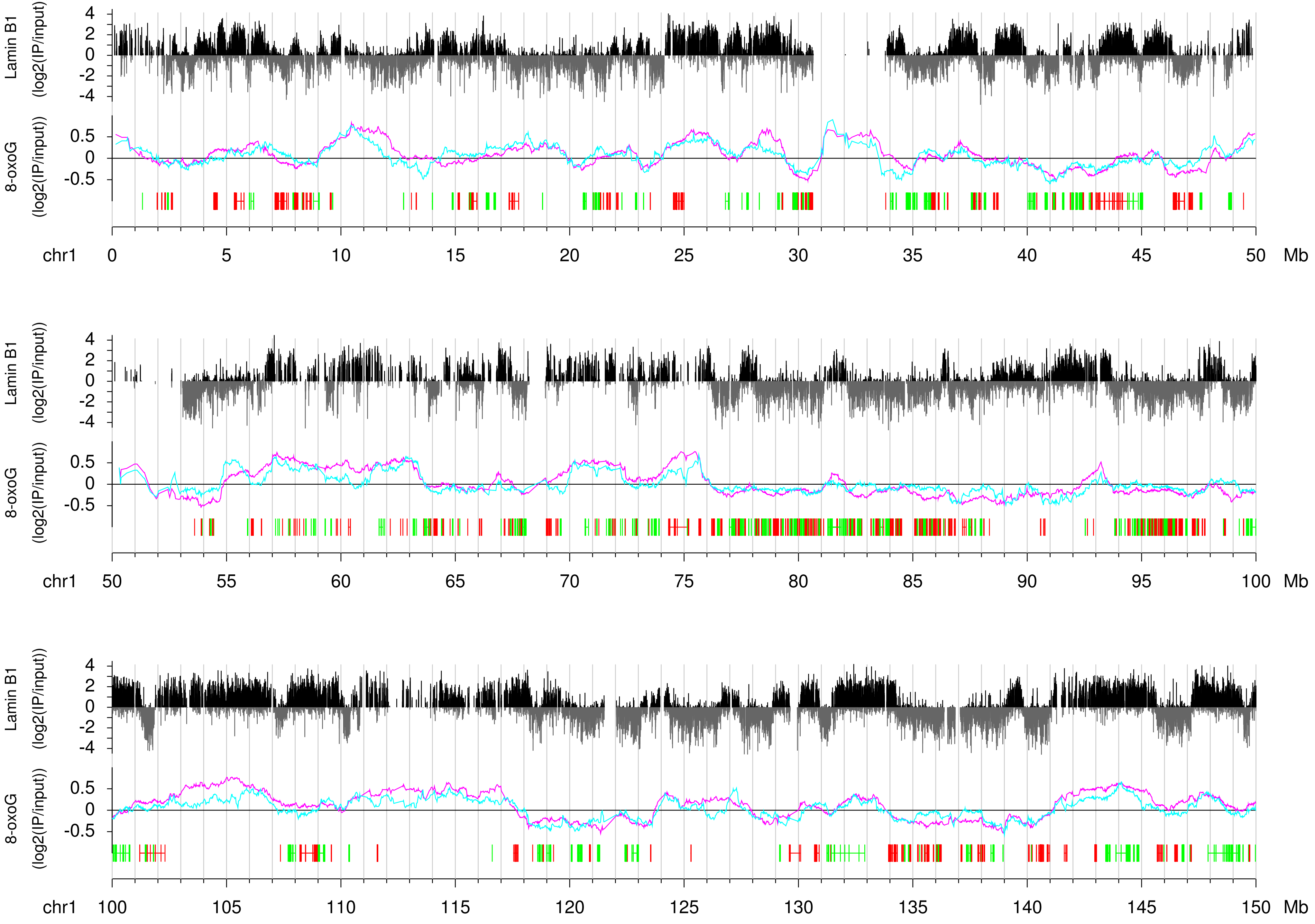
Lamin B1
(log2(IP/input))
8-oxoG
(log2(IP/input))
chr1
Mb
0
5
10
15
20
25
30
35
40
45
50
55
60
65
70
75
80
85
90
95
100
105
110
115
120
125
130
135
140
145
150

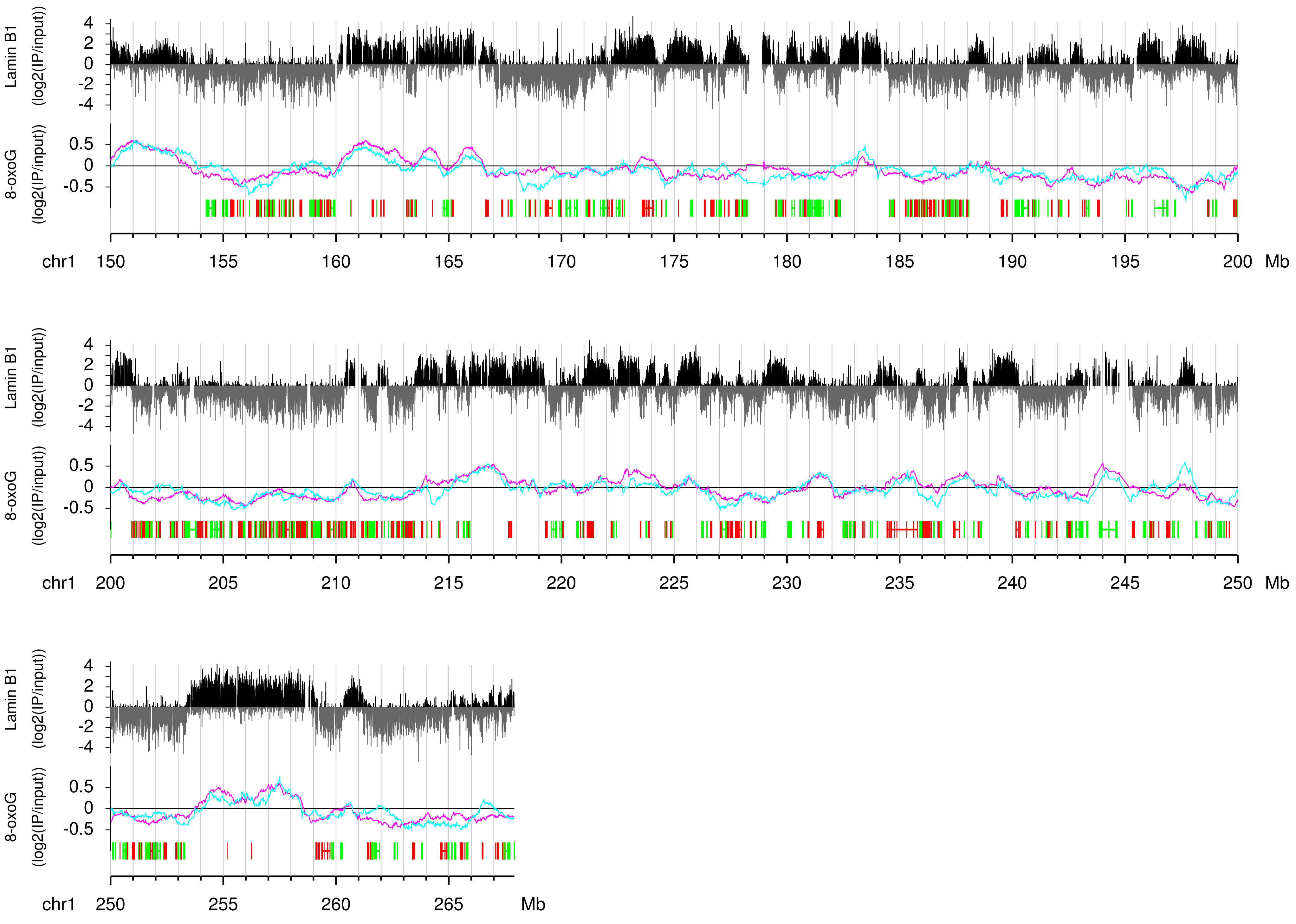
Lamin B1
(log2(IP/input))
4
2
0
-2
-4
8-oxoG
(log2(IP/input))
0.5
0
-0.5
chr1
150
155
160
165
170
175
180
185
190
195
200
Mb
200
205
210
215
220
225
230
235
240
245
250
250
255
260
265

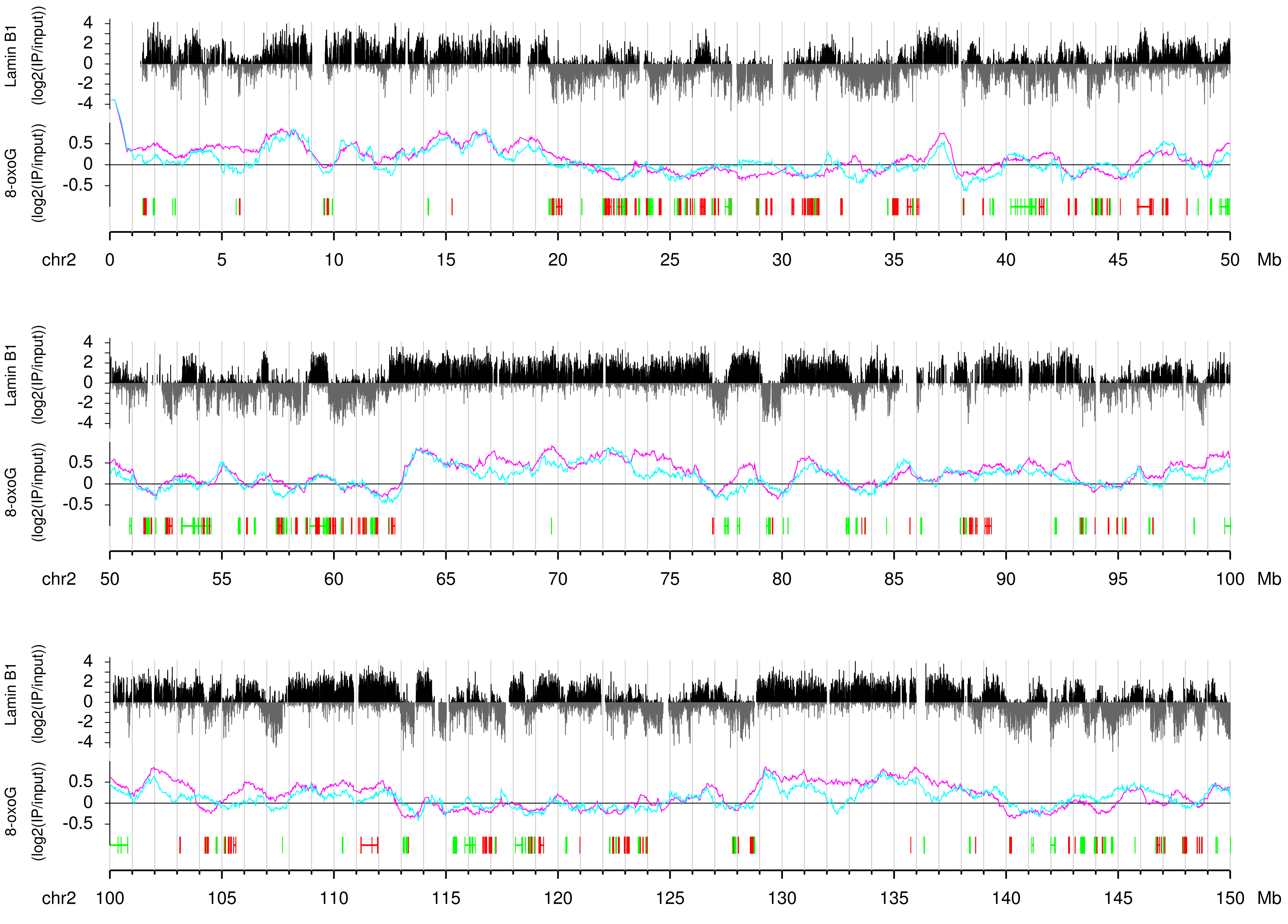
Lamin B1
(log2(IP/input))
8-oxoG
(log2(IP/input))
chr2
Mb
0
5
10
15
20
25
30
35
40
45
50
55
60
65
70
75
80
85
90
95
100
105
110
115
120
125
130
135
140
145
150
4
2
0
-2
-4
0.5
0
-0.5

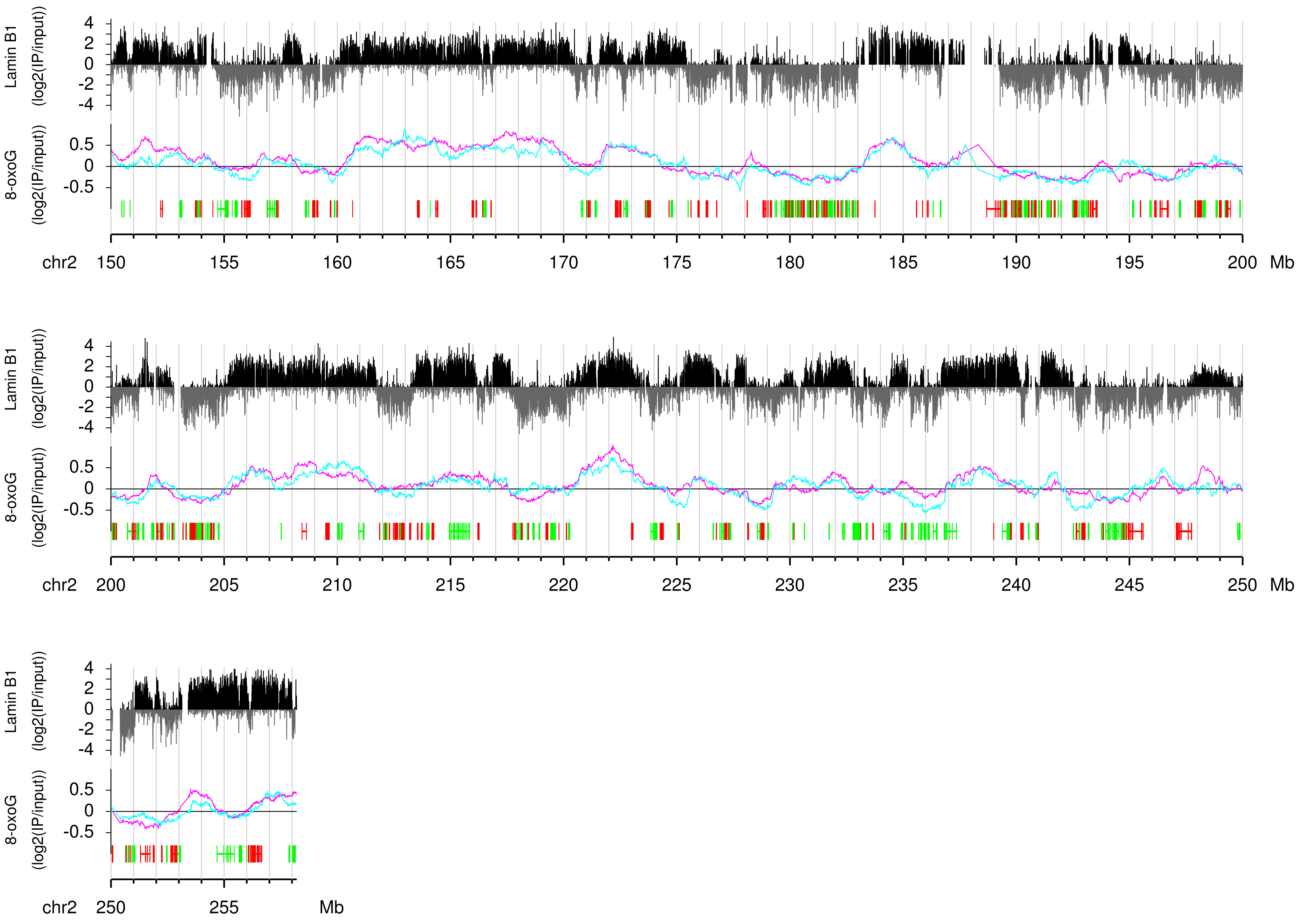
Lamin B1 (log2(IP/input))
4 2 0 -2 -4
8-oxoG (log2(IP/input))
0.5 0 -0.5
chr2 150 155 160 165 170 175 180 185 190 195 200 Mb
Lamin B1 (log2(IP/input))
4 2 0 -2 -4
8-oxoG (log2(IP/input))
0.5 0 -0.5
chr2 200 205 210 215 220 225 230 235 240 245 250 Mb
Lamin B1 (log2(IP/input))
4 2 0 -2 -4
8-oxoG (log2(IP/input))
0.5 0 -0.5
chr2 250 255 Mb

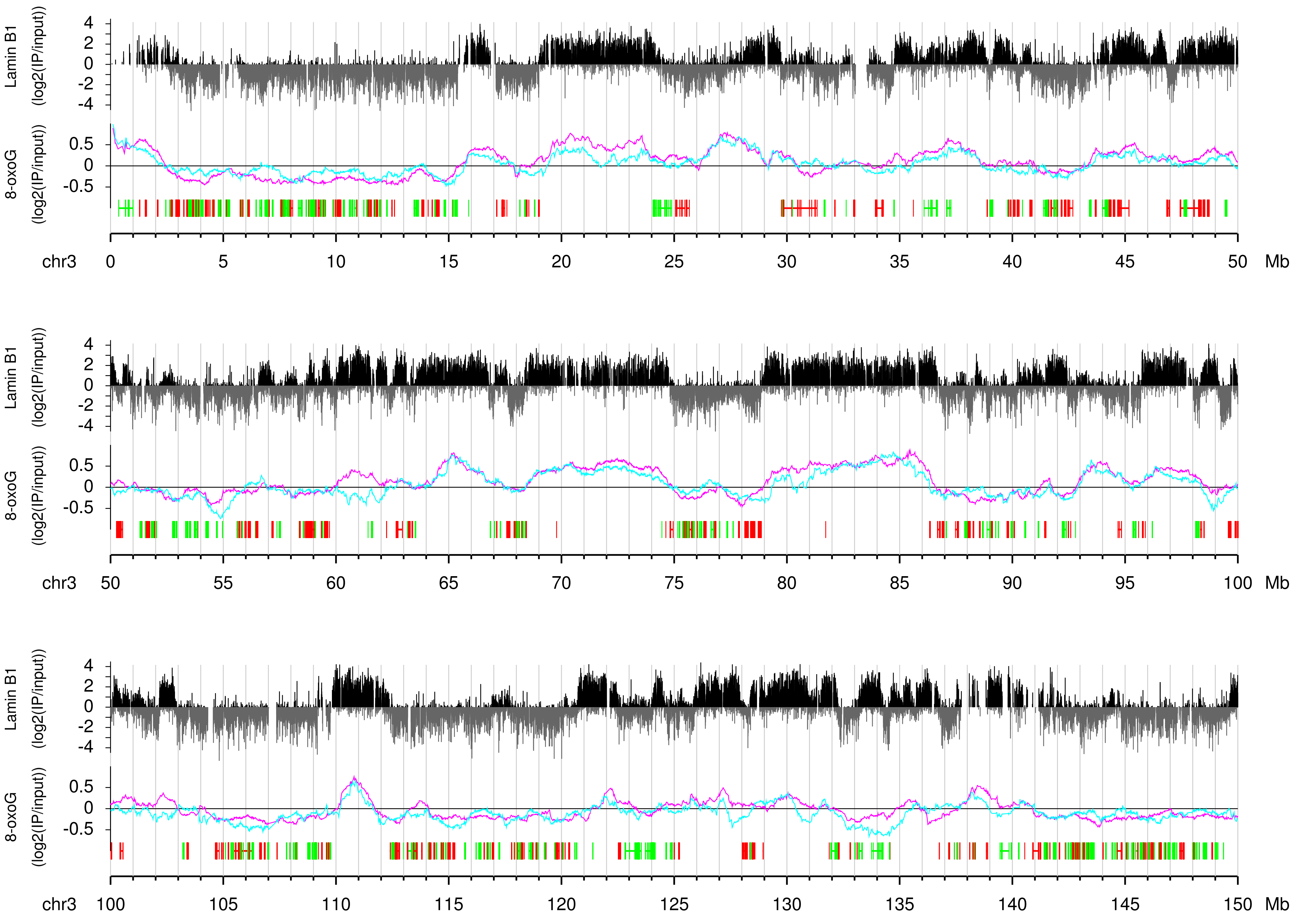
Lamin B1
(log2(IP/input))
8-oxoG
(log2(IP/input))
4
2
0
-2
-4
0.5
0
-0.5
chr3
0
5
10
15
20
25
30
35
40
45
50
Mb
50
55
60
65
70
75
80
85
90
95
100
100
105
110
115
120
125
130
135
140
145
150

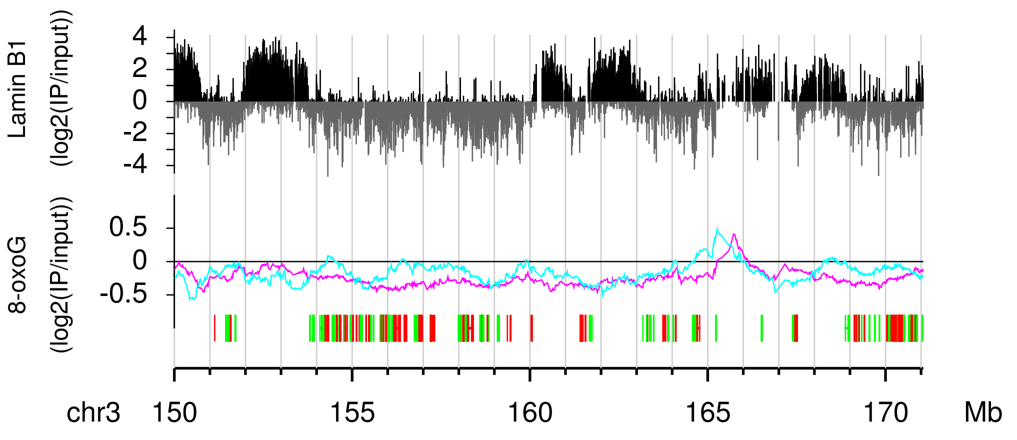
Lamin B1
(log2(IP/input))
8-oxoG
(log2(IP/input))
chr3
Mb

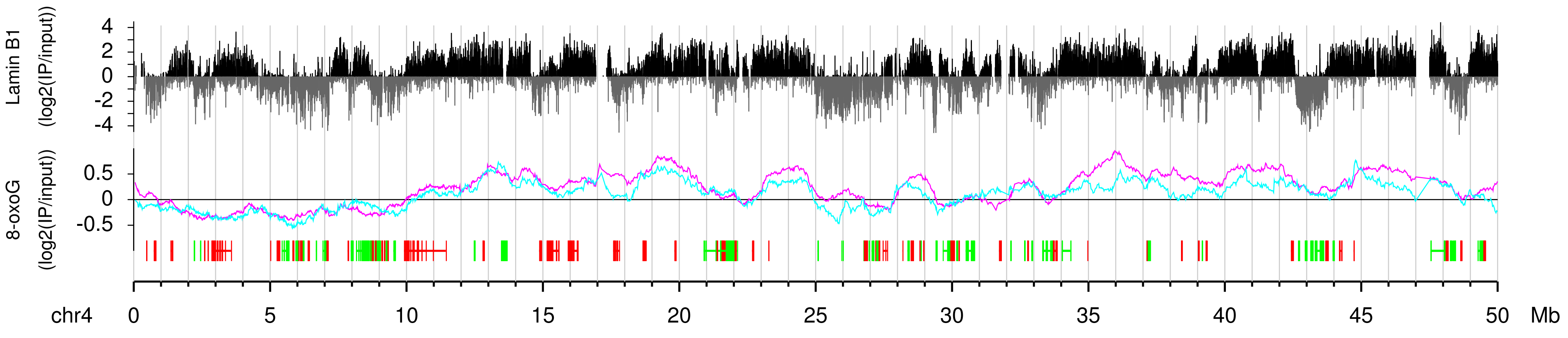
Lamin B1
(log2(IP/input))
8-oxoG
(log2(IP/input))
chr4
Mb

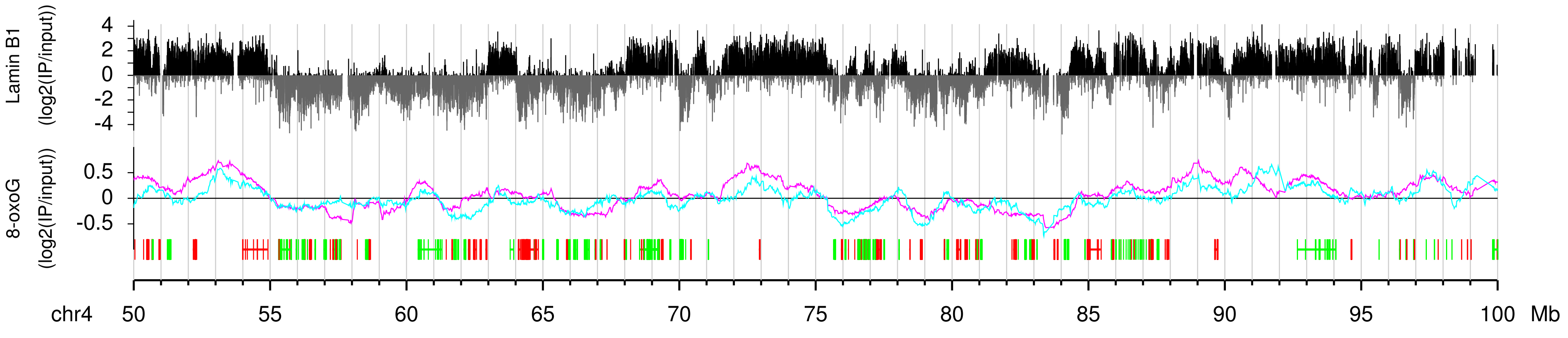
Lamin B1
(log2(IP/input))
8-oxoG
(log2(IP/input))
chr4
Mb

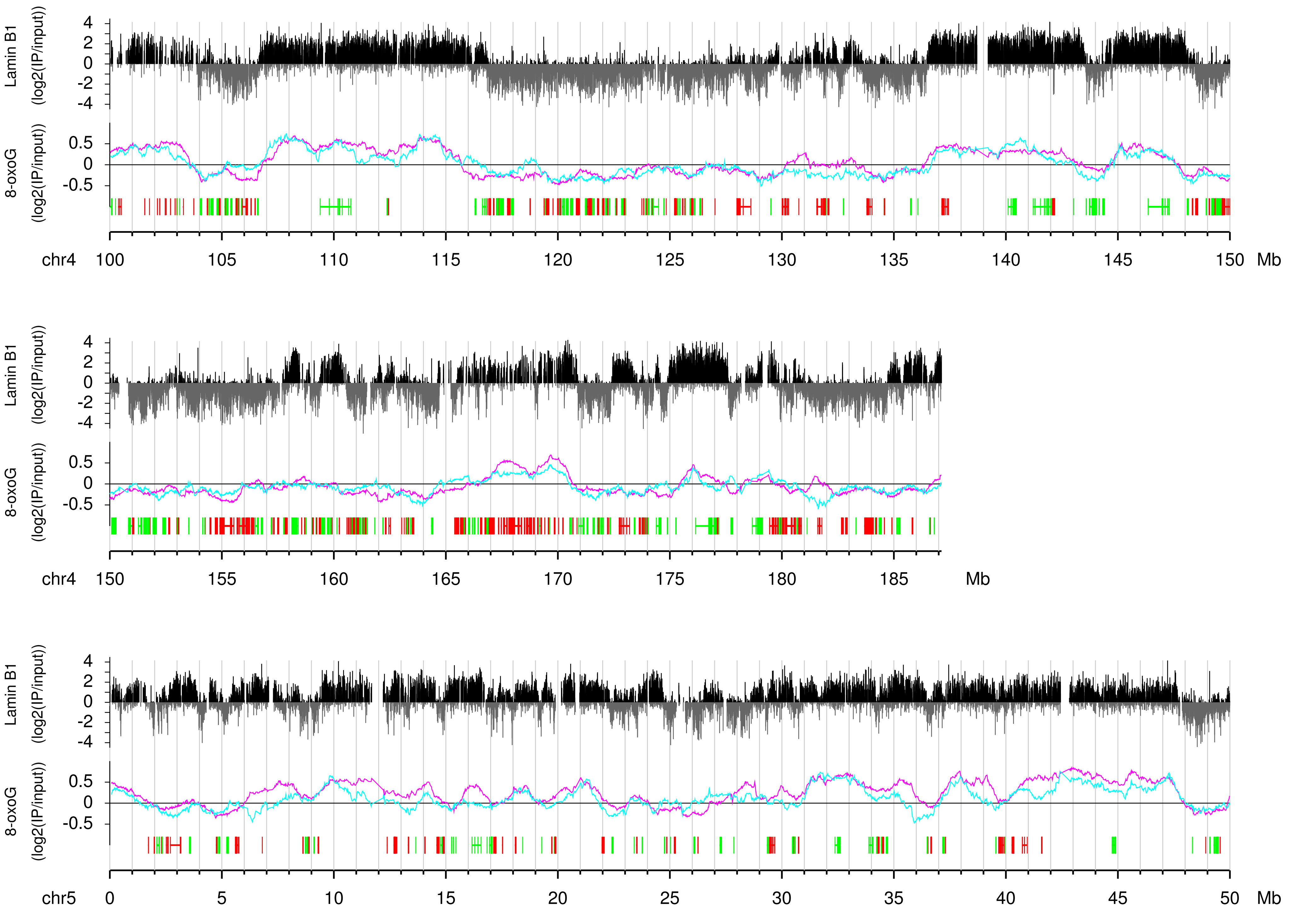
Lamin B1
(log2(IP/input))
8-oxoG
(log2(IP/input))
chr4
Mb
chr4
Mb
chr5
Mb

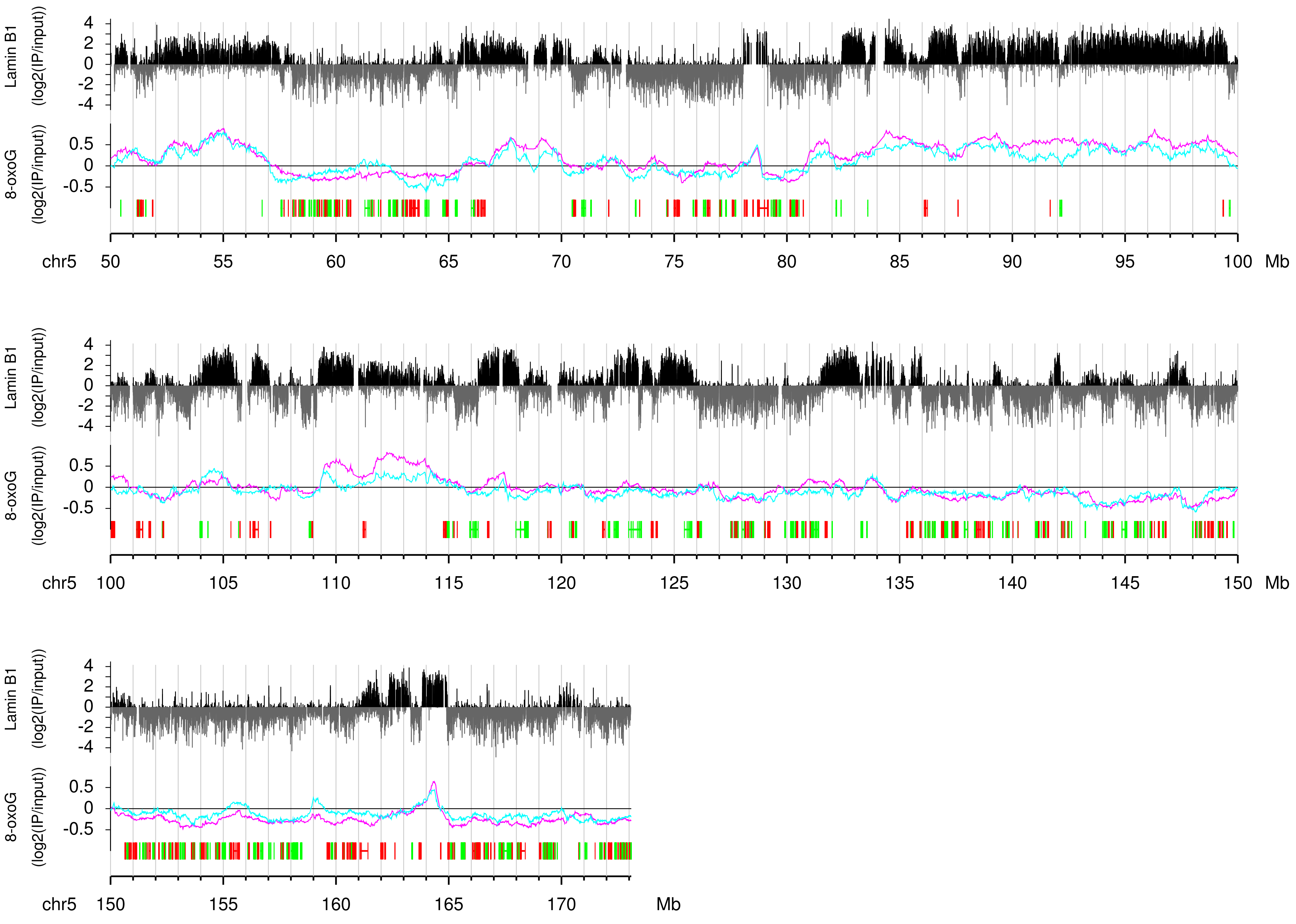
Lamin B1
(log2(IP/input))
8-oxoG
(log2(IP/input))
chr5
Mb

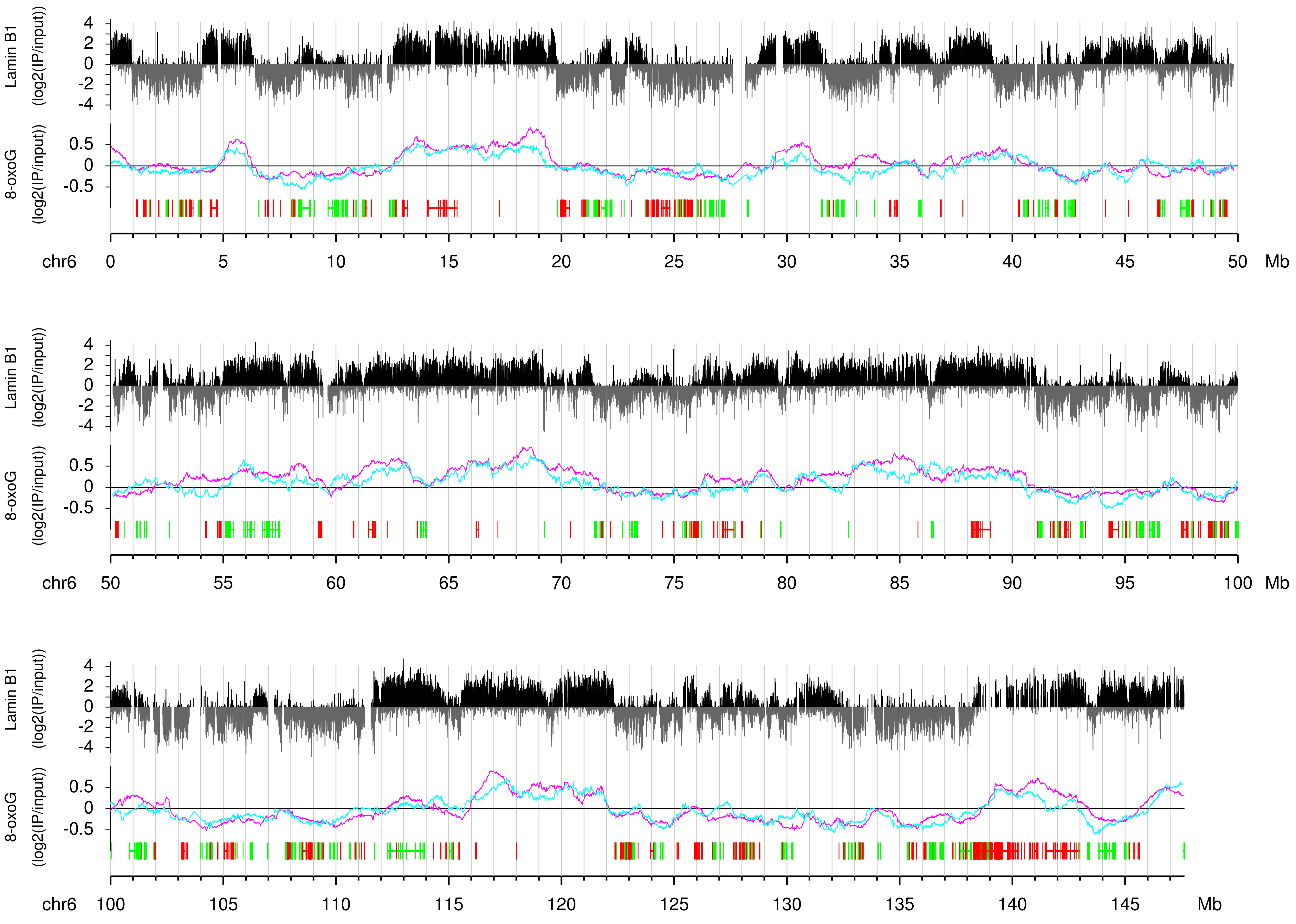
Lamin B1
(log2(IP/input))
8-oxoG
(log2(IP/input))
chr6
Mb

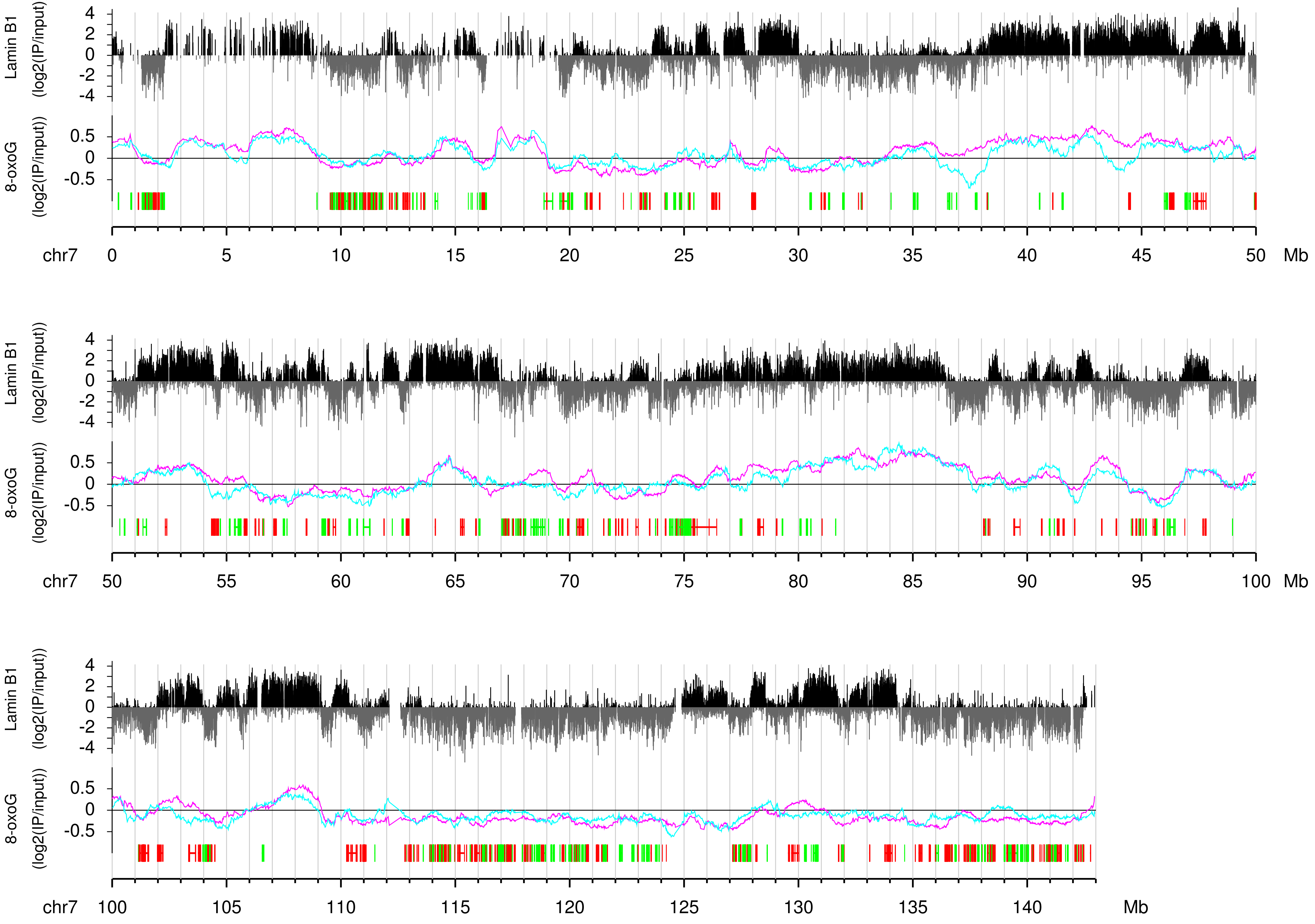
Lamin B1
(log2(IP/input))
8-oxoG
(log2(IP/input))
chr7
Mb

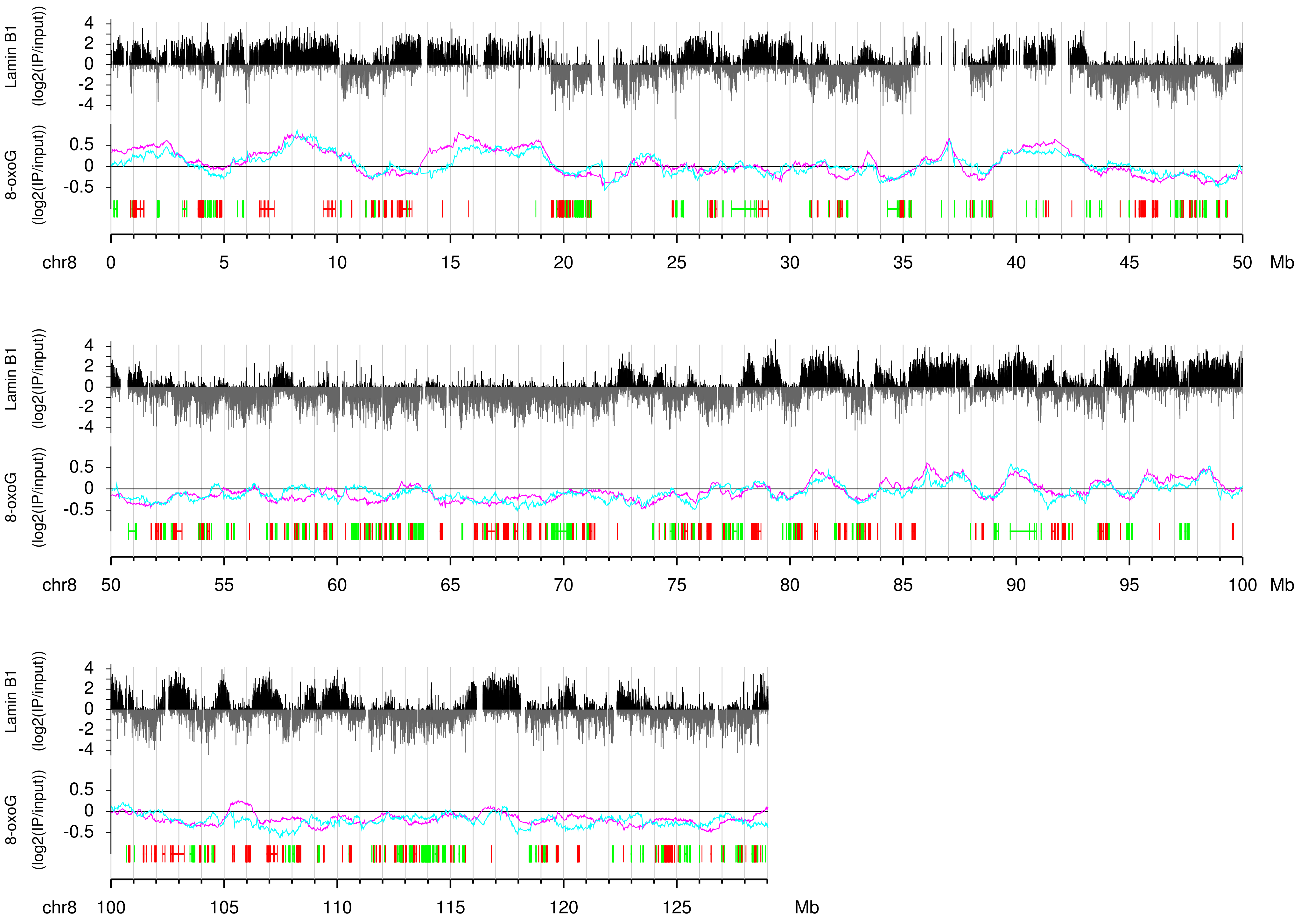
Lamin B1
(log2(IP/input))
8-oxoG
(log2(IP/input))
chr8
Mb
Lamin B1
(log2(IP/input))
8-oxoG
(log2(IP/input))
chr8
Mb
Lamin B1
(log2(IP/input))
8-oxoG
(log2(IP/input))
chr8
Mb

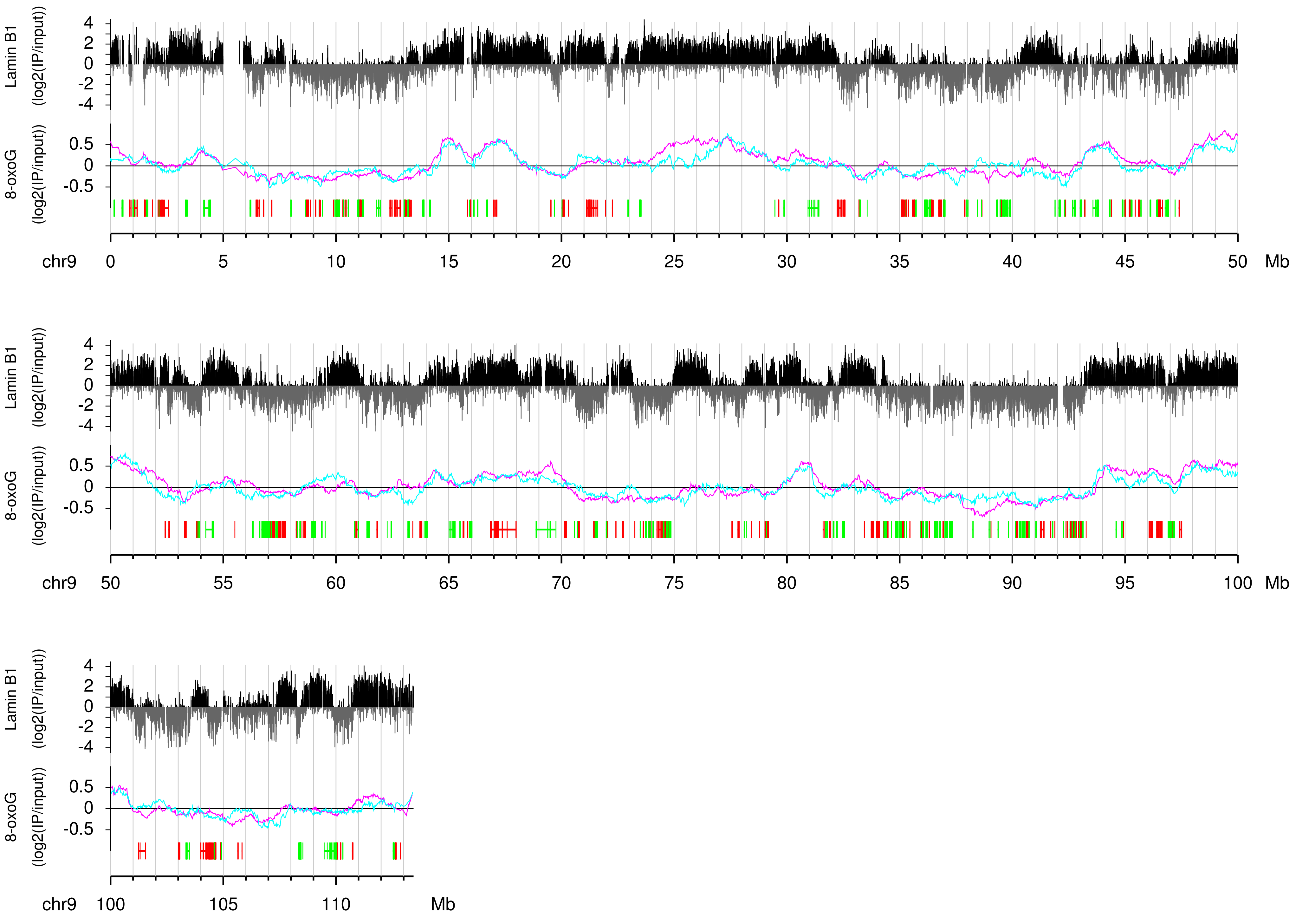
Lamin B1
(log2(IP/input))
8-oxoG
(log2(IP/input))
chr9
Mb

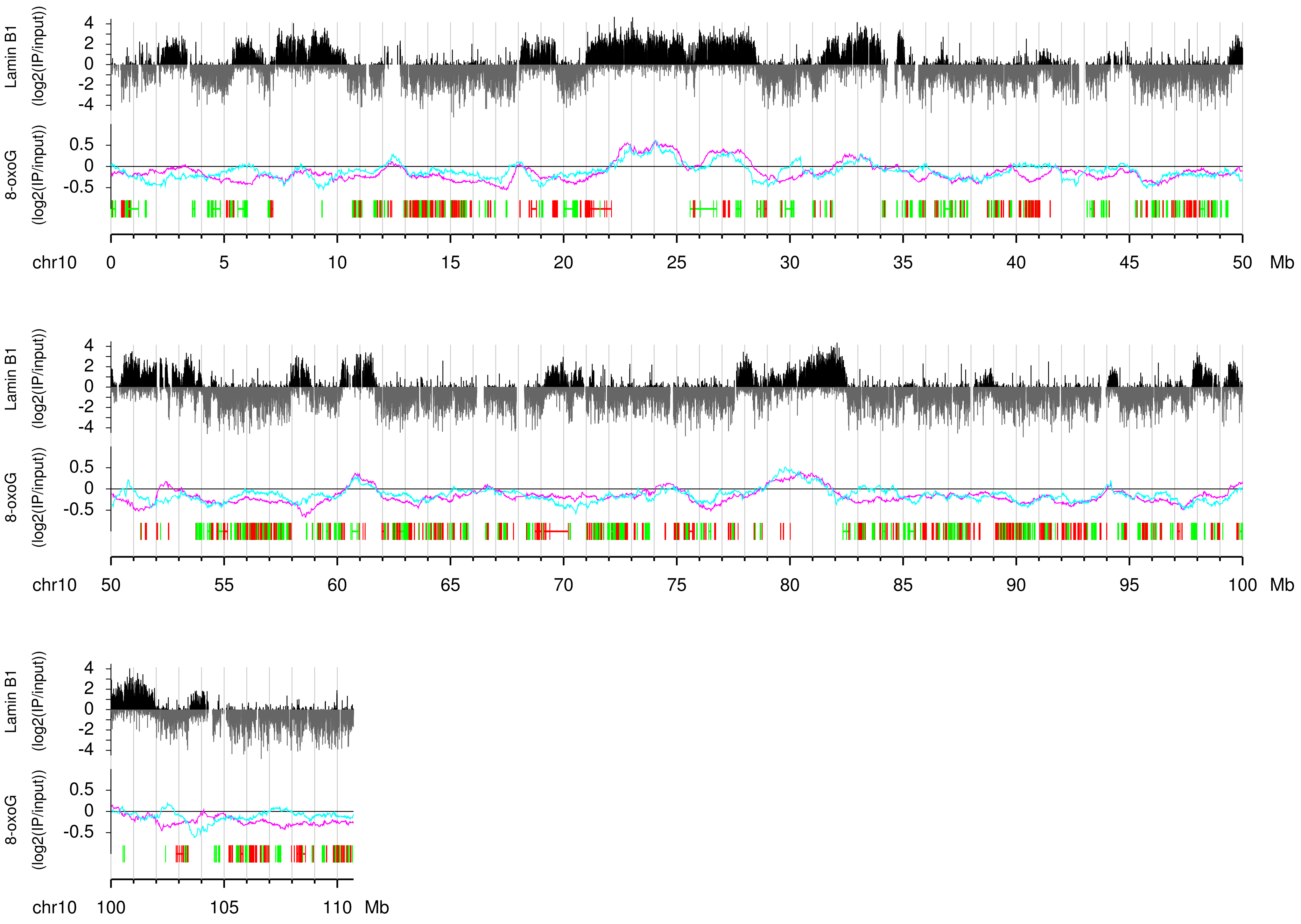
Lamin B1 (log2(IP/input))
8-oxoG (log2(IP/input))
chr10
0 5 10 15 20 25 30 35 40 45 50 Mb
50 55 60 65 70 75 80 85 90 95 100 Mb
100 105 110 Mb

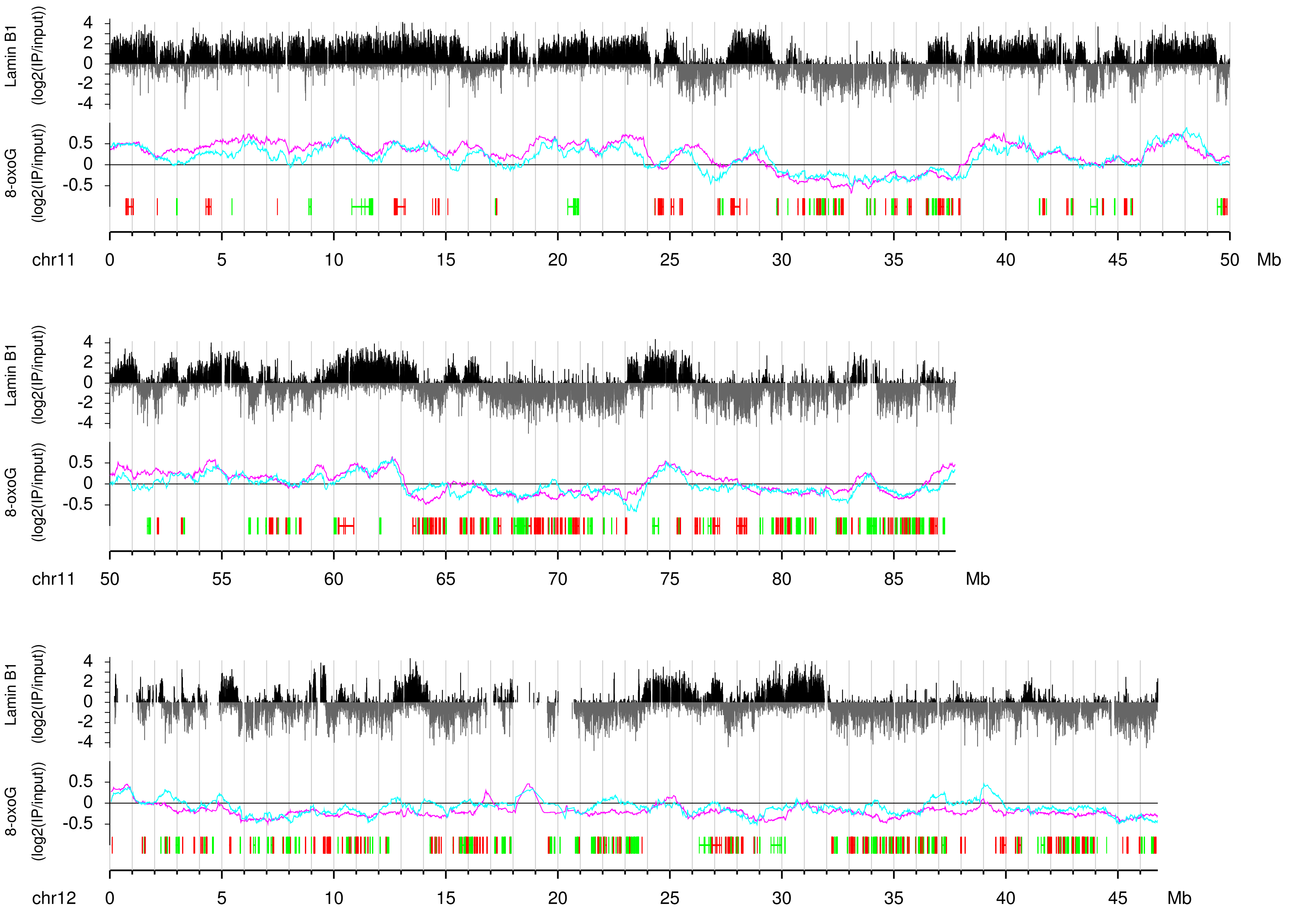

Lamin B1
(log2(IP/input))
4
2
0
-2
-4
8-oxoG
(log2(IP/input))
0.5
0
-0.5
chr11
0
5
10
15
20
25
30
35
40
45
50
Mb
chr11
50
55
60
65
70
75
80
85
Mb
chr12
Mb

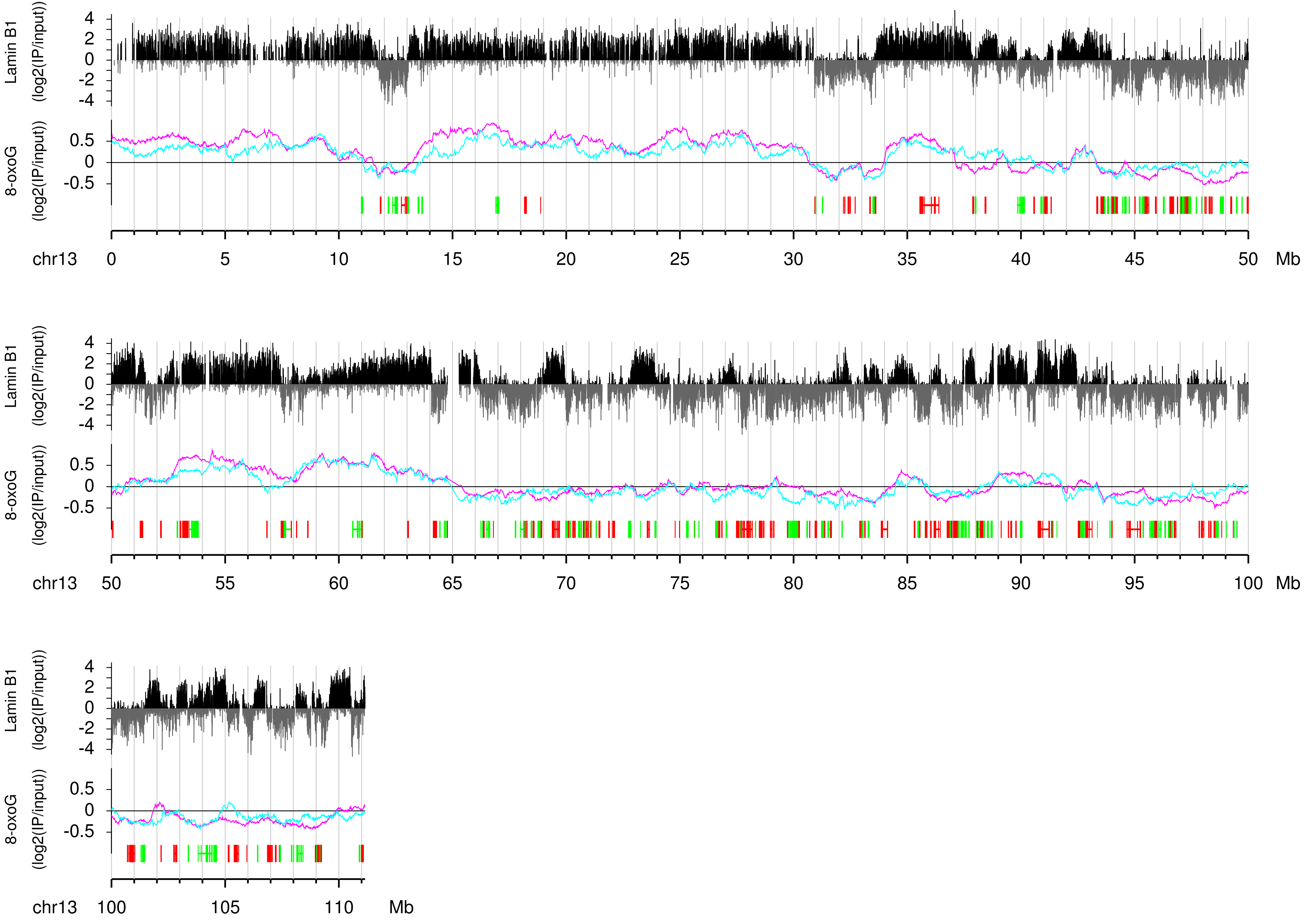

Lamin B1
(log2(IP/input))
8-oxoG
(log2(IP/input))
chr13
Mb

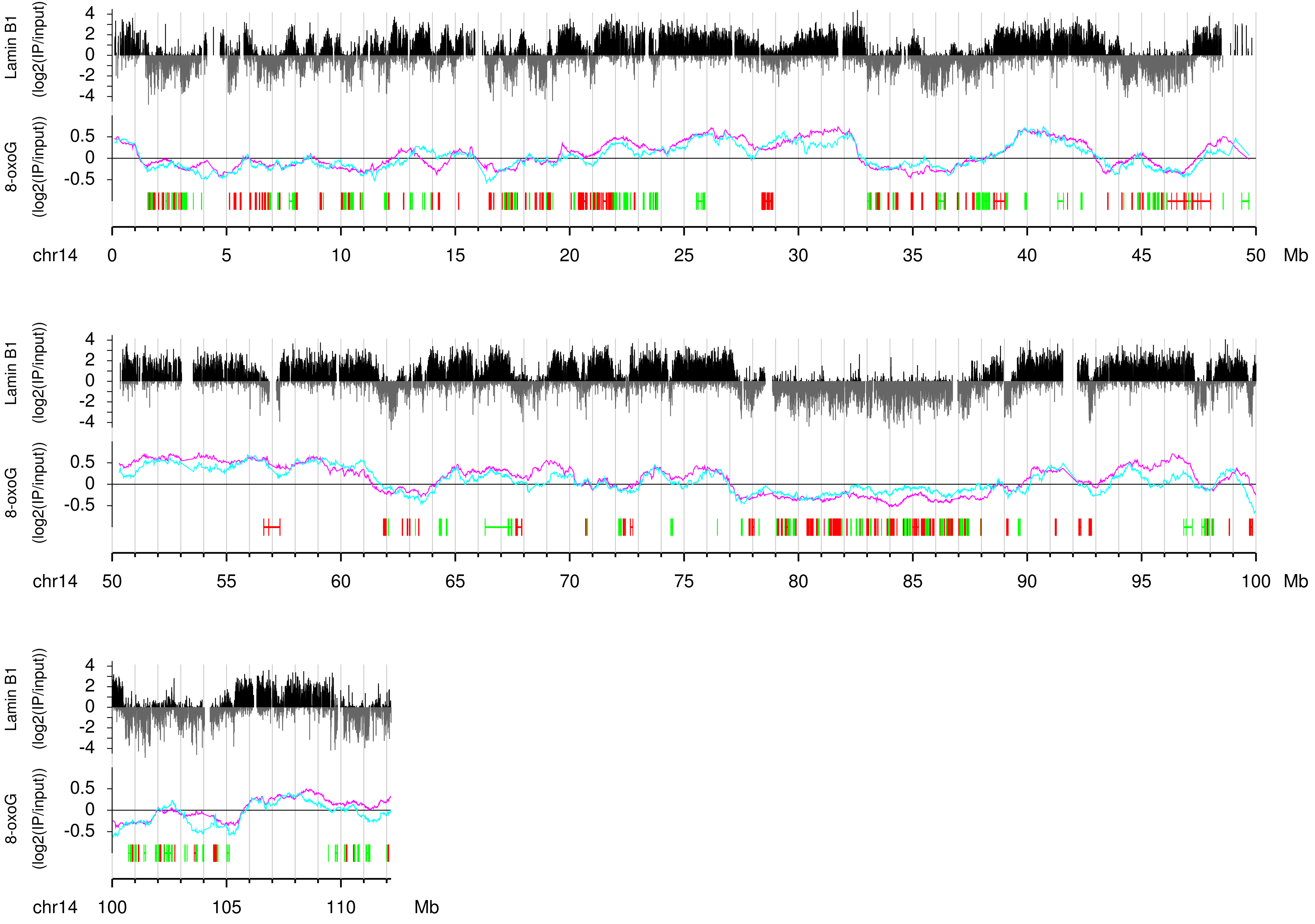
Lamin B1
(log2(IP/input))
8-oxoG
(log2(IP/input))
chr14
Mb

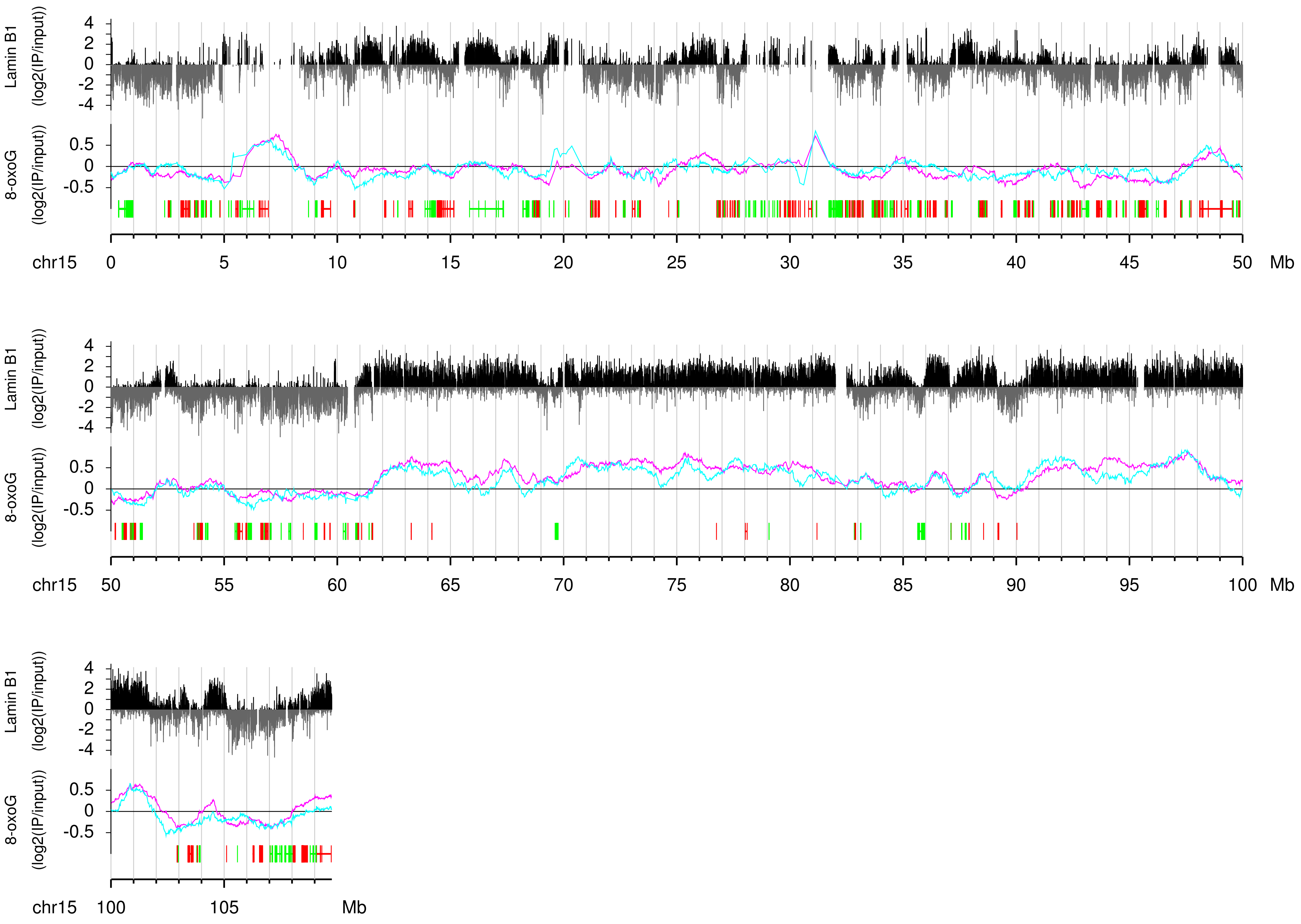
Lamin B1
(log2(IP/input))
8-oxoG
(log2(IP/input))
4
2
0
-2
-4
0.5
0
-0.5
chr15
0
5
10
15
20
25
30
35
40
45
50
Mb
50
55
60
65
70
75
80
85
90
95
100
100
105

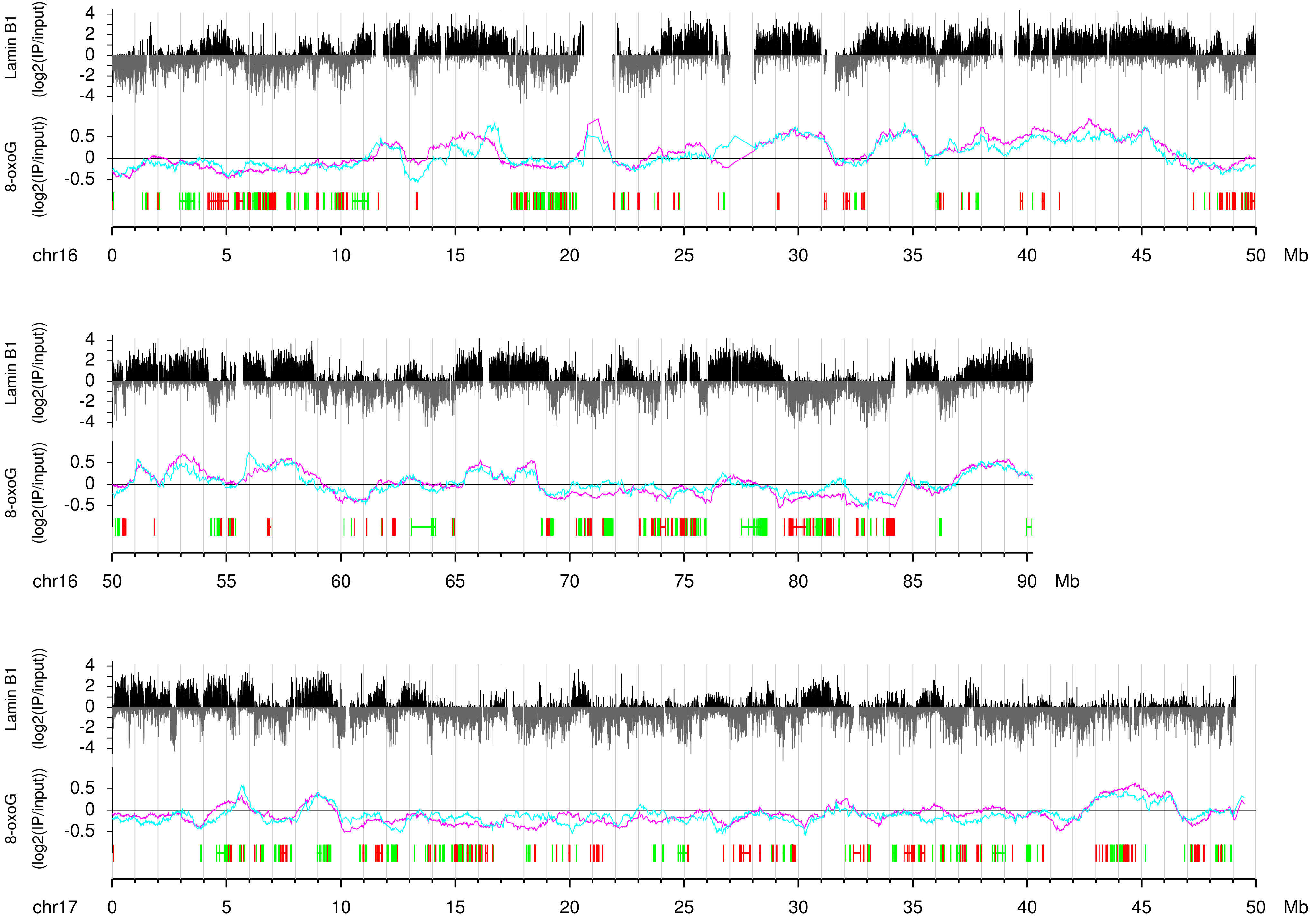
Lamin B1
(log2(IP/input))
8-oxoG
(log2(IP/input))
chr16
chr17
Mb

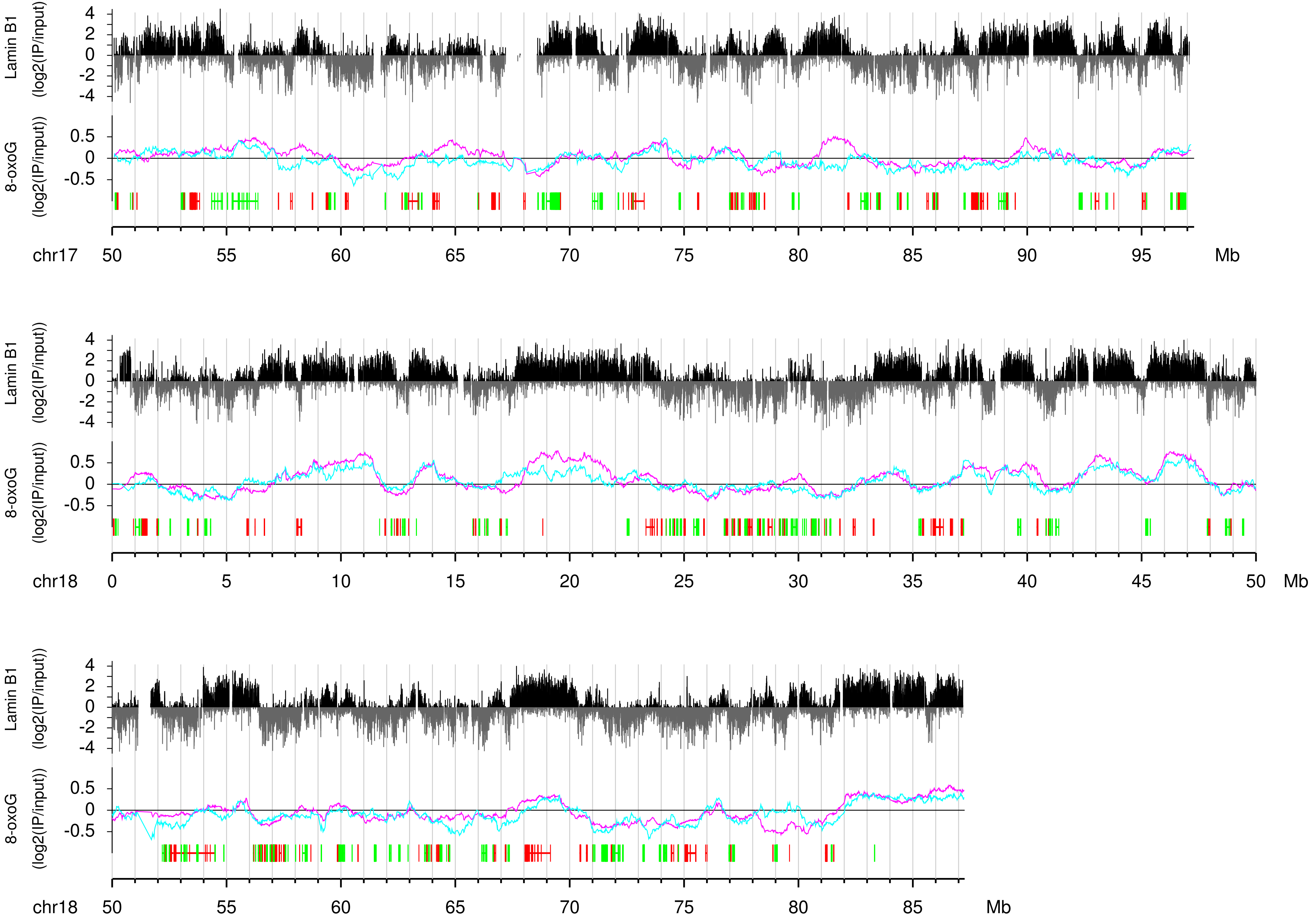
Lamin B1
(log2(IP/input))
8-oxoG
(log2(IP/input))
chr17
Mb
chr18
Mb
chr18
Mb

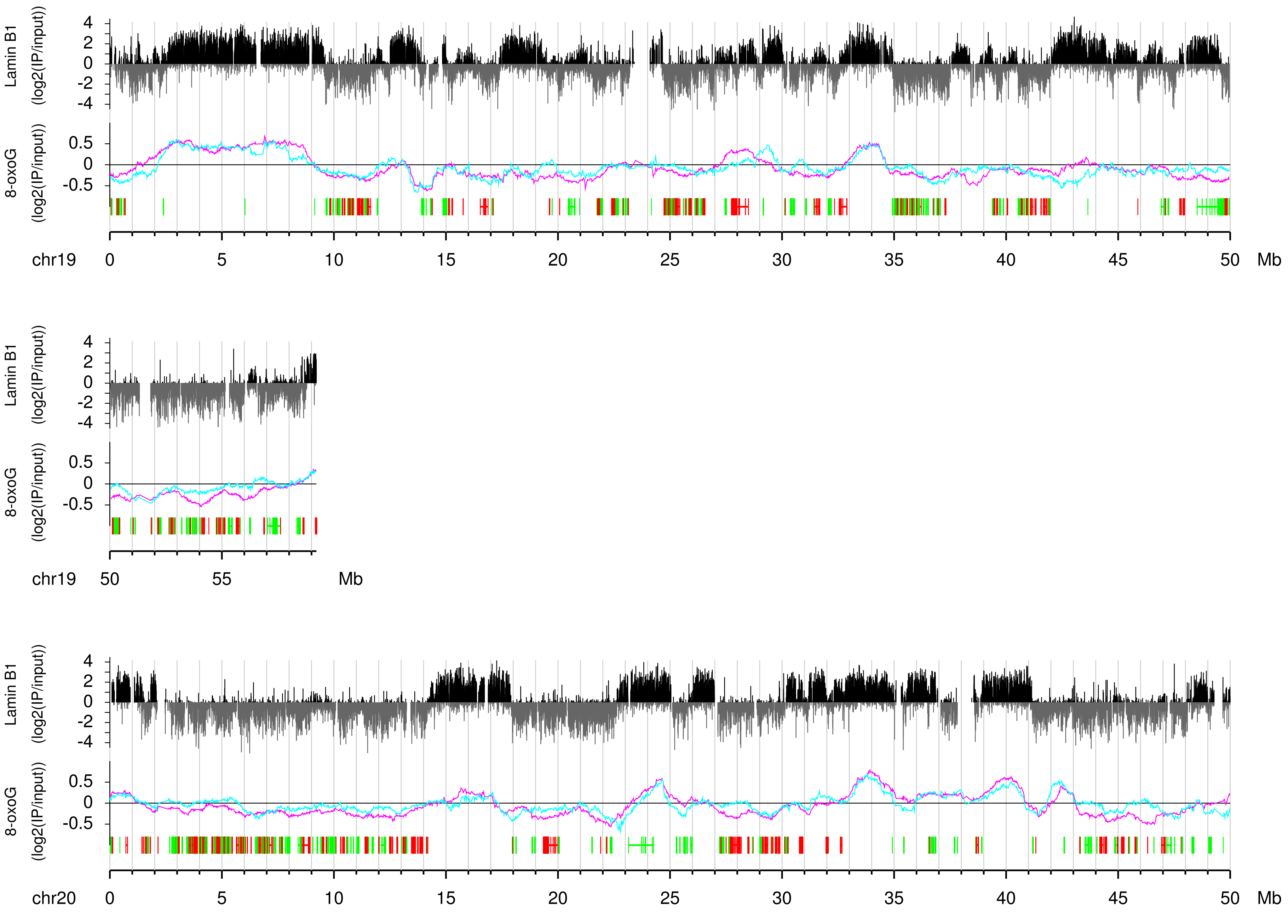

Lamin B1
(log2(IP/input))
8-oxoG
(log2(IP/input))
chr19
Mb
chr19
Mb
Lamin B1
(log2(IP/input))
8-oxoG
(log2(IP/input))
chr20
Mb

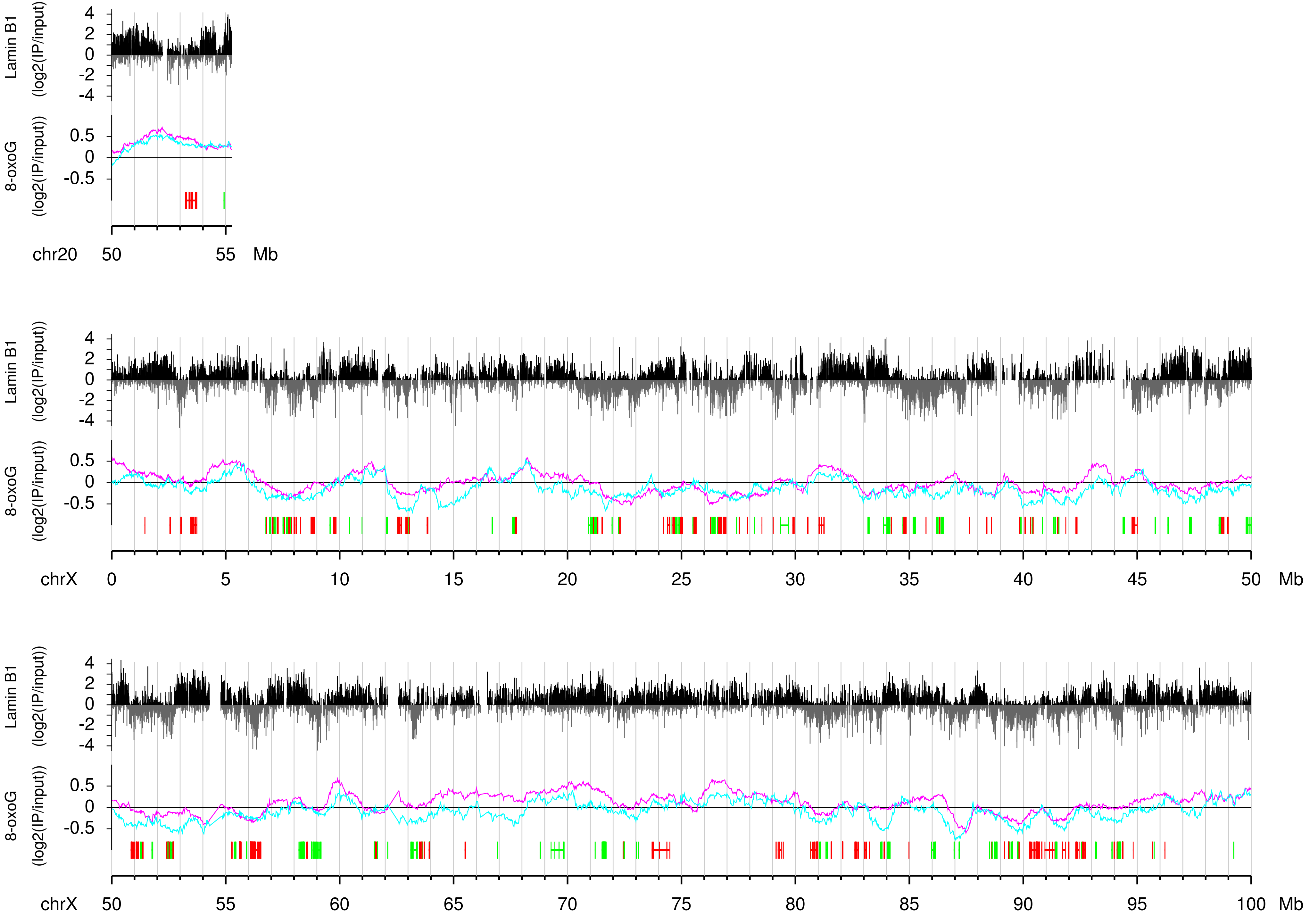
Lamin B1
(log2(IP/input))
8-oxoG
(log2(IP/input))
4
2
0
-2
-4
0.5
0
-0.5
chr20
50
55
Mb
chrX
0
5
10
15
20
25
30
35
40
45
50
60
65
70
75
80
85
90
95
100

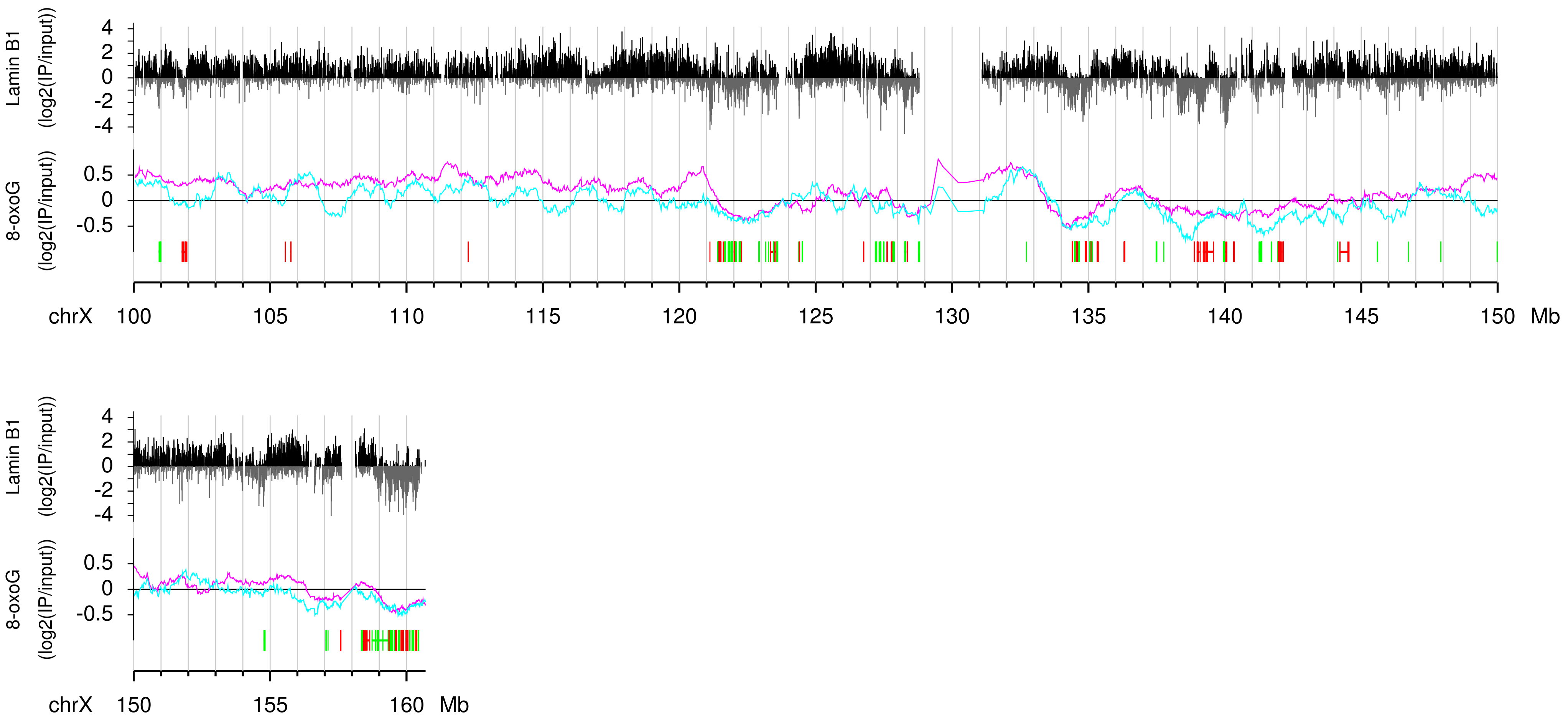


Figure S2. 8-oxoG profile (middle track) and the profile of LADs (upper track) along the chromosomes. The profile of 8-oxoG was calculated by averaging the intensities over a 1-Mb sliding window. Two biological replicates are shown in pink and cyan lines. Gene structures are shown below the 8-oxoG profile. The directionality of the genes, i.e., forward and reverse orientation, is shown in green and red, respectively.

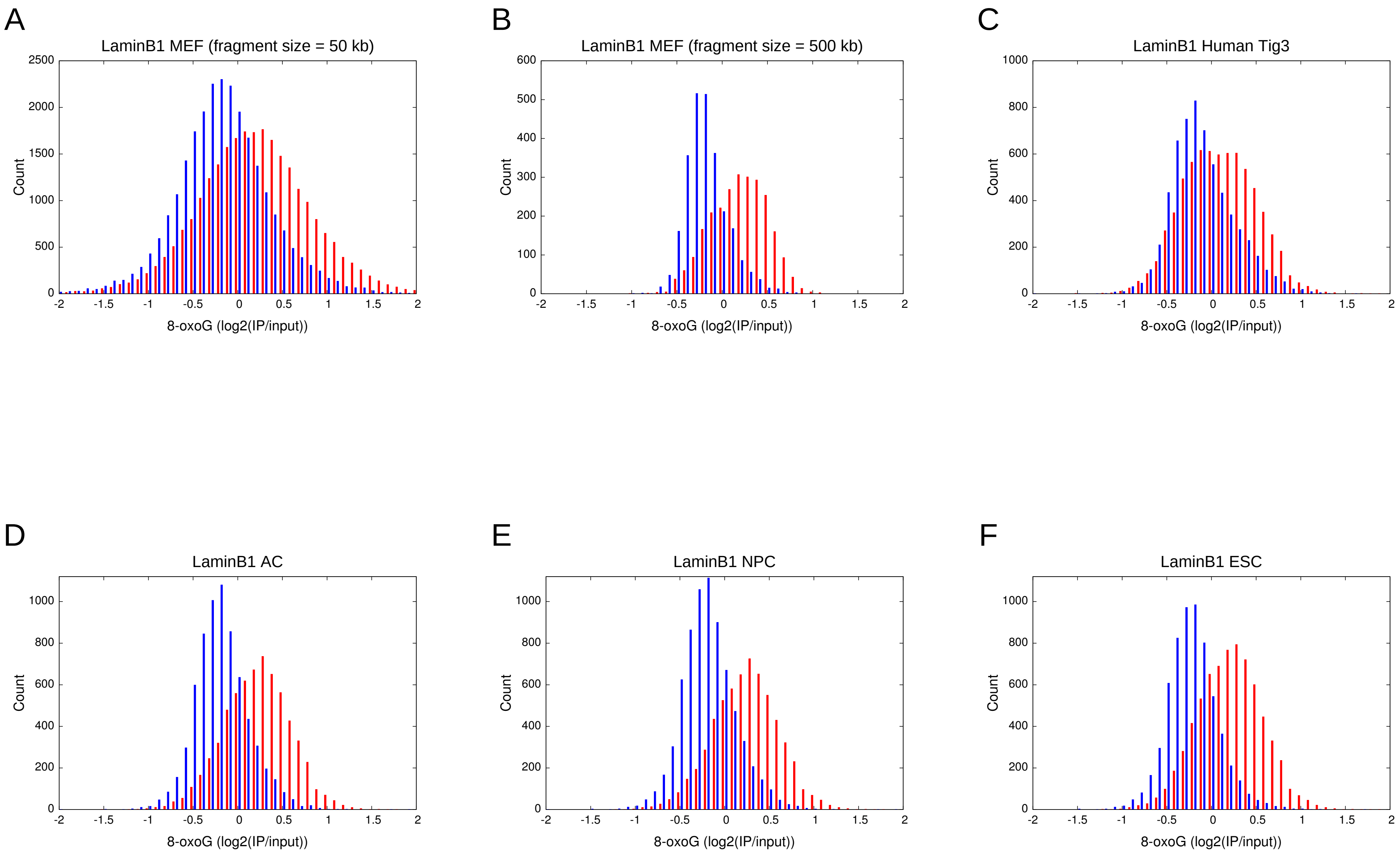


Figure S3. Histograms of averaged values of 8-oxoG in genomic fragments. The fragment size for averaging is 200 kb, unless otherwise stated. The fragments are grouped into two subsets using the averaged lamin B1 value of 0 as cutoff. Sets with high and low content of lamin B1 are shown in red and blue, respectively. (A and B) mouse embryonic fibroblasts, (C) human Tig3 lung fibroblasts, (D) mouse astrocytes, (E) mouse neural progenitor cells, and (F) mouse embryonic stem cells.
